# Coxsackievirus-Induced miR-21 Disrupts Cardiomyocyte Interactions via the Downregulation of Intercalated Disk Components

**DOI:** 10.1371/journal.ppat.1004070

**Published:** 2014-04-10

**Authors:** Xin Ye, Huifang Mary Zhang, Ye Qiu, Paul J. Hanson, Maged Gomaa Hemida, Wei Wei, Pamela A. Hoodless, Fanny Chu, Decheng Yang

**Affiliations:** 1 Department of Pathology and Laboratory Medicine, University of British Columbia, The Centre for Heart Lung Innovation, St. Paul's Hospital, Vancouver, British Columbia, Canada; 2 Terry Fox Laboratory, British Columbia Cancer Agency, Vancouver, British Columbia, Canada; Oregon Health and Science University, United States of America

## Abstract

Intercalated disks (ICDs) are substantial connections maintaining cardiac structures and mediating signal communications among cardiomyocytes. Deficiency in ICD components such as desmosomes, fascia adherens and gap junctions leads to heart dysfunction. Coxsackievirus B3 (CVB3) infection induces cardiac failure but its pathogenic effect on ICDs is unclear. Here we show that CVB3-induced miR-21 expression affects ICD structure, i.e., upregulated miR-21 targets YOD1, a deubiquitinating enzyme, to enhance the K48-linked ubiquitination and degradation of desmin, resulting in disruption of desmosomes. Inhibition of miR-21 preserves desmin during CVB3 infection. Treatment with proteasome inhibitors blocks miR-21-mediated desmin degradation. Transfection of miR-21 or knockdown of YOD1 triggers co-localization of desmin with proteasomes. We also identified K108 and K406 as important sites for desmin ubiquintination and degradation. In addition, miR-21 directly targets vinculin, leading to disturbed fascia adherens evidenced by the suppression and disorientation of pan-cadherin and α-E-catenin proteins, two fascia adherens-components. Our findings suggest a new mechanism of miR-21 in modulating cell-cell interactions of cardiomyocytes during CVB3 infection.

## Author Summary

Coxsackievirus B3 (CVB3) is one of most common causes of heart inflammation and failure. However, the mechanism by which CVB3 induces cardiac damage has not been fully elucidated. Particularly, the involvement of microRNAs (miRNAs), a family of small RNAs controlling the progression of a wide range of diseases, in CVB3 infection is still unclear. These small RNAs are essential to understand the CVB3-caused heart muscle cell injury and have great potential to serve therapeutic purposes. Here, we systematically analyzed the miRNA changes during CVB3 infection and found that miR-21 is increased by viral infection. We further demonstrated that the CVB3-induced miR-21 triggers heart muscle cell damage by interfering with the cell-cell interactions. miR-21 suppresses the levels of components in cell-cell interactions by either promoting the degradation of those proteins or directly inhibiting the protein production. Inhibition of miR-21 can reduce the host injury caused by CVB3 infection. Our findings will shed new lights on the pathogenesis of CVB3-induced heart failure.

## Introduction

microRNAs (miRNAs) are endogenous gene regulators functioning through targeting messenger RNAs (mRNAs) [1]. Their capability of targeting several genes simultaneously enables their vast involvement in physiological and pathological conditions [2], including cardiac dysfunctions and viral infections [3], [4]. Among these small RNAs, miR-21 is one of the most essential ones due to its wide involvement in development and diseases [5].

miR-21 acts as a pivotal pillar in controlling the pathogenesis of cardiovascular diseases [6]. This is evidenced by the fact that miR-21 is abundant in cardiovascular system and dramatically altered during the development of the diseases such as cardiac hypertrophy [7], fibrosis [8] and myocardial infarction [9]. However, the functional role of miR-21 directly related to cardiomyocyte physiology, is controversial. Thum et al. showed that neither enhancement nor suppression of miR-21 affects the morphology, size or number of cardiomyocytes in primary culture [10], while another group demonstrated that miR-21 induces cardiomyocyte outgrowth [7]. Cardioprotective roles of miR-21 have been suggested by several studies using hypertrophy [11] or ischemia models [12] in which miR-21 attenuates hypertrophic growth and inhibits the cardiomyocyte death but genetic knock-out experiments and locked nucleic acid-mediated inhibition of miR-21 expression suggest no essential role of miR-21 in pathological myocardium remodeling [13].

miR-21 is also actively involved in viral infections. Epstein-Barr virus (EBV) infection stimulates miR-21 expression, resulting in B-cell transformation [14]. Hepatitis C virus (HCV) induces miR-21 expression to evade host immune system [15]. Coxsackievirus B3 (CVB3) is a major cause of myocarditis, an infectious heart diseases characterized by inflammation and damage of myocardium, accounting for ∼20% of sudden unexpected death in youth and infants [16], [17]. We and others have shown that CVB3 infection induces significant changes in host miRNA expression profiles, which in turn modulate viral infection or contribute to the progression of the disease [18]–[21]. For miR-21, its altered expression and functional role during CVB3 infection have been studied by a number of groups while the results on the altered expression are inconsistent. Jin He and co-workers found that miR-21 was downregulated in CVB3 infected mice, resulting in the upregulation of PDCD4 and cardiomyocyte apoptosis [15]. However, two other groups showed that CVB3 infection upregulates miR-21 in mice [19], [22]. The latter one further indicated that miR-21 promotes the differentiation of Th-17 (Helper T) cells that produce interleukin-17 (IL-17), leading to inflammation while the detailed mechanism has not been identified [22].

Intercalated disks (ICDs) are essential cell-cell connections consisting of desmosomes, fascia adherens and gap junctions. They are critical components for cardiac functions and their disruption leads to heart dysfunction [23]. Desmosomes anchor the cell membranes to the intermediate filaments to maintain the proper cell-cell connections [24]. Adherens junctions link the cell membranes to cytoskeleton components like actin [25]. Gap junctions channel the electronic and metabolic signals among the cardiomyocytes [26]. Disorganization of these structures results in pathological heart conditions such as hypertrophy, dilated cardiomyopathy and arrhythmia, symptoms similar to CVB3 induced cardiac failure [23], [27]. miR-21 enhances the gap junctions by targeting SPRY2 and triggering the redistribution of Connexin 43 and β-catenin [7]. However, whether and how miR-21 regulates desmosome or adherens junctions in cardiomyocytes is entirely unknown. To address these issues, we analyzed the miRNA expression profiles in CVB3 infected mouse hearts and cultured mouse and human cardiomyocytes, and found that miR-21 was robustly increased by CVB3 infection. Functional characterization found that virus-induced increase of miR-21 expression injures cardiomyocytes by two ways: i) disturbing desmosome structures by targeting YOD1, a deubiquitinating enzyme, to enhance the ubiquitin-mediated degradation of desmin proteins and ii) interrupting fascia adherens organization by directly targeting VCL and suppressing its protein translation.

## Results

### Microarray Analysis of miRNA Expression Profiles and Confirmation of miR-21 Upregulation by CVB3 Infection

**Figure 1:**
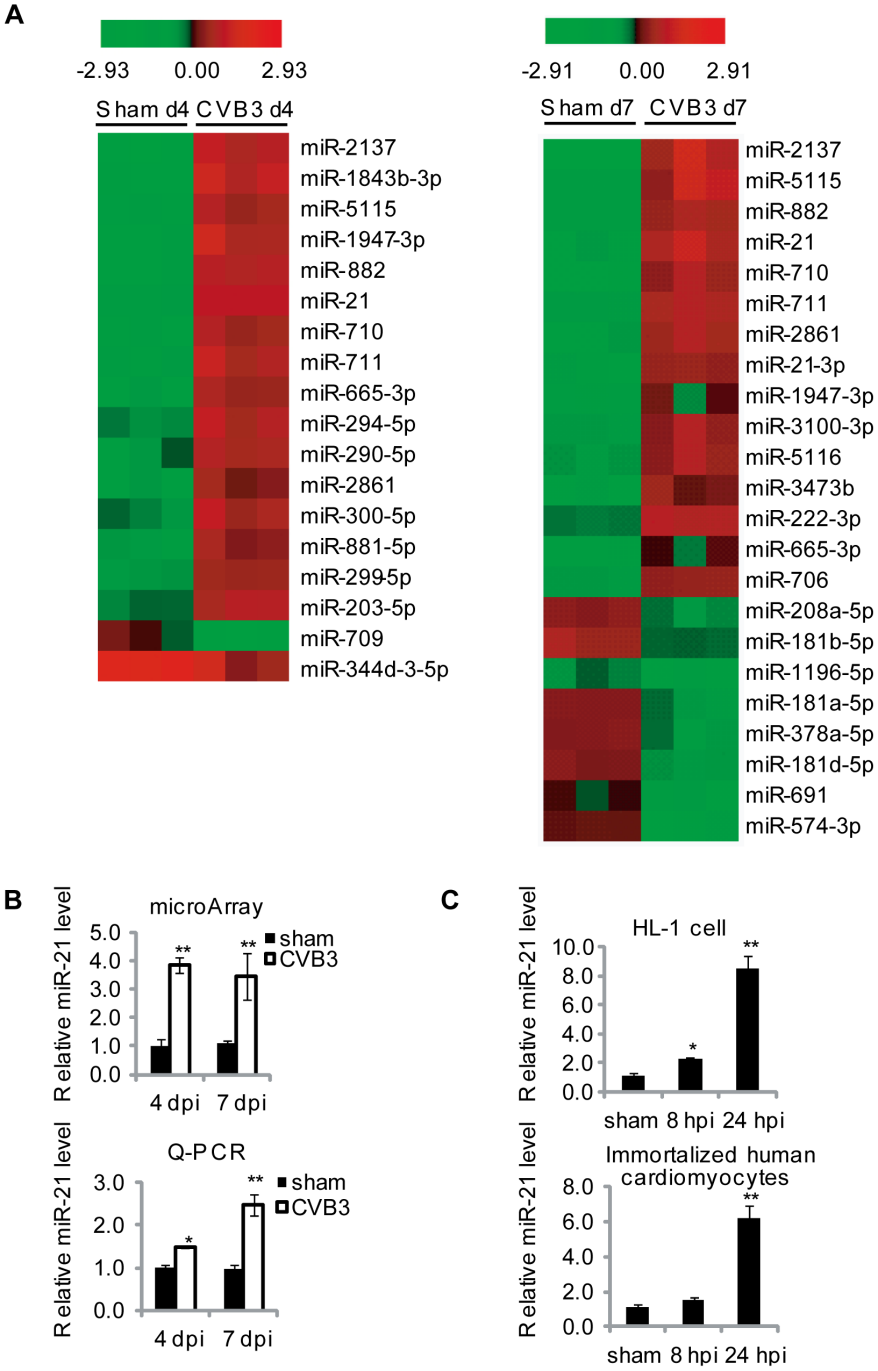
CVB3 infection upregulates miR-21 expression. (**A**) Partial heat map of differentially regulated miRNAs (*p*<0.05, fold change >2) in CVB3 infected A/J mouse hearts compared with sham infected ones. The color refers to the Log2 value of miRNA expression levels. (**B**) Quantification of miR-21 expression detected by microarray analysis and q-RT-PCR of tissues with viral myocarditis. All data were normalized to U6 RNA and then further normalized to that of sham controls at 4 dpi. (**C**) miR-21 induction by CVB3 infection *in vitro*. HL-1 mouse cardiomyocytes or immortalized human cardiomyocytes were infected by CVB3 as indicated. “*” stands for *p*<0.05 and “**” means *p*<0.01. For microarray data, n = 3 and for q-RT-PCR, n = 5.

To identify miRNA candidates regulating CVB3-induced viral myocarditis, we performed microarray analysis of miRNA expression profiles using RNAs isolated from CVB3-infected mouse hearts, an established viral myocarditis model. We first confirmed the myocarditis occurrence by observing leukocyte infiltration and cardiomyocyte necrosis, which demonstrated that severe myocarditis appeared at both 4-day post infection (4 dpi) and 7 dpi (Figure S1A in Text S1). VP-1 expression was detected as an indicator for successful infection (Figure S1B in Text S1). Based on these confirmations of infection and disease presence, total RNAs isolated from these tissues were employed to conduct microarray analysis. miRNAs with more than 2-fold changes compared with controls were selected as top miRNA candidates. As shown in the heat map in Figure 1A, 16 miRNAs were upregulated and 2 were downregulated at 4 dpi compared with the control group; while 15 miRNAs were increased and 8 were decreased at 7 dpi. Among all these miRNAs, eight appeared in the lists of miRNAs identified at both 4 and 7 dpi, indicating their potential functions in viral myocarditis. We also conducted quantitative reverse transcriptase PCR (q-RT-PCR) to confirm the alterations of some miRNA candidates including miR-21, miR-203-3p, miR-222-3p and miR-574-3p (Figure 1B, S1C in Text S1). The results were consistent with the microarray analysis data. We chose miR-21 for further investigation due to its critical role in cardiac diseases. Both microarray and q-RT-PCR confirmed a 2–4 fold increase in CVB3 infected mouse hearts compared with the control (Figure 1B). The q-RT-PCR results showed that the induction of miR-21 expression in CVB3 infected group is greater at 7 dpi than at 4 dpi; while such difference was not obvious in the microarray data, indicating that q-RT-PCR may be more sensitive than microarray analysis in this type of analysis. To further confirm the upregulation of miR-21 during CVB3 infection *in vitro*, HL-1 mouse cardiomyocytes and immortalized human cardiomyocytes were used to detect miR-21 expression levels after CVB3 infection by q-RT-PCR. Compared to sham-infected control, CVB3 infection triggered a 5–10 folds upregulation in the infected cells (Figure 1C). We then infected HL-1 cells with UV-irradiated CVB3 to determine whether active viral replication is required for miR-21 upregulation. Q-PCR results showed that UV-inactivated CVB3 failed to stimulate miR-21 (Figure S2 in Text S1), indicating that viral replication is required for the induction of miR-21. These data demonstrated that CVB3 infection induces upregulation of miR-21 expression in cardiomyocytes *in vitro* and *in vivo*.

### miR-21 Downregulates Desmin Levels and Disrupts Desmosome Organization during CVB3 Infection

We first evaluated the effect of miR-21 on CVB3 replication. HL-1 cells and immortalized human cardiomyocytes were transfected with 10 nM of miR-21 mimics or 50 nM of miR-21 inhibitor (21-in). miR-CL, a miRNA mimic control with a scrambled sequence, was used as a negative control for miR-21. We also transfected the cells with miR-362, a non-relevant miRNA as another negative control. According to bioinformatic analysis using TargetScan [28], miR-362 only shared 4 predicted targets with miR-21 in the total 110 potential targets and none of the 4 targets (ARHGEF12, BCL11A, PURB and TNRC6B) were known to be involved in the signal pathways in this study. miRNA inhibitor control (CL-in) was used as a negative control for 21-in. As shown in Figure S3A in Text S1, transfection of miR-21 mimics led to a ∼10-fold increase in miR-21 level compared with transfection of miR-CL. This increased level is similar to that induced by CVB3 infection. In addition, 21-in reduced miR-21 expression by ∼90% compared with the control. Viral replication levels were measured by detecting viral protein VP-1 and performing viral plaque assay. Neither miR-21 nor 21-in exhibited any effect on VP-1 level or viral plaque numbers in both HL-1 cells and human cardiomyocytes (Figure S4 in Text S1), indicating that miR-21 may not affect CVB3 replication.

**Figure 2:**
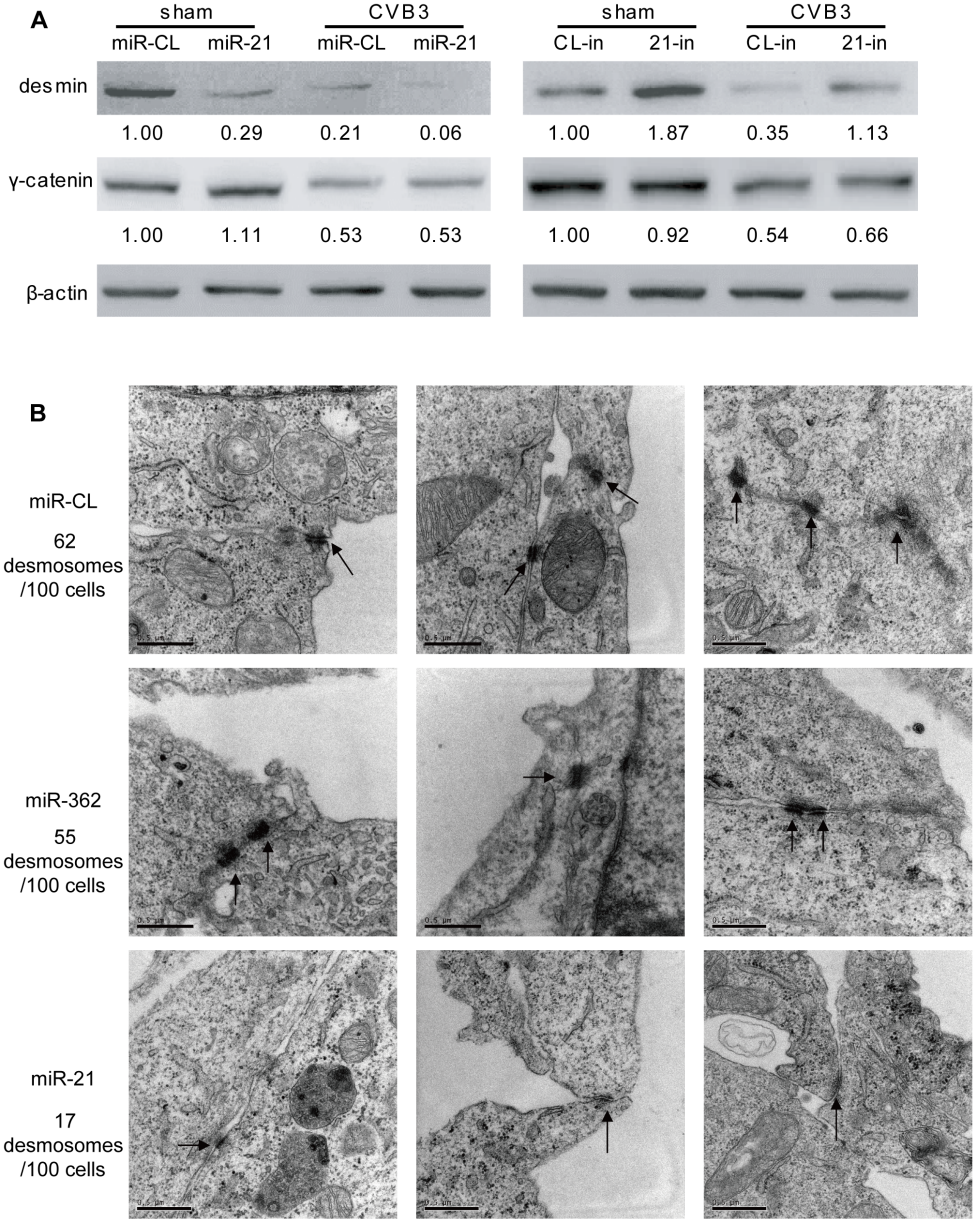
miR-21 downregulates desmin and disrupts desmosome structures. (**A**) miR-21 expression reduces desmin levels during CVB3 infection. HL-1 cells were transfected and infected as indicated. Desmin, γ-catenin and β-actin were detected by WB. The intensities of the bands were measured by using ImageJ and the signal ratios were listed below. (**B**) miR-21 expression disrupts desmosome structure. HL-1 cells transfected with miR-CL, miR-362 or miR-21 mimics were subjected to EM analysis on desmosome number and structure. Three representative views were listed for each sample and desmosomes were indicated with black arrows. For each sample, 100 cells were analyzed and the numbers of desmosomes observed were indicated. Magnification: 37000×. Bar: 0.5 µm.

As mentioned above, miR-21 is involved in regulating cell-cell connections in cardiomyocytes, we thus further explored the effect of miR-21 upregulation on desmosome structure by detecting the expression of γ-catenin (plakoglobin), a major intracellular component of desmosome, and desmin, the intermediate filaments closely associating with desmosomes to maintain its structure and function. In sham-infected controls, transfection of miR-21 mimics inhibited desmin expression by ∼70% (from 1.00 to 0.29) while 21-in increased desmin expression by ∼90% (from 1.00 to 1.87). In the miR-CL transfected cells, a robust reduction (∼80%, from 1.00 to 0.21) in desmin expression was found in CVB3 infected cells compared with the sham-infected control. Transfection of miR-21 mimics enhanced virus-induced suppression of desmin expression while treatment of 21-in substantially attenuated the downregulation of desmin during CVB3 infection (Figure 2A). On the contrary, miR-21 showed no significant influence on γ-catenin level. The decreased levels of desmin protein may affect desmosome formation and its structures. This speculation was verified by electronic microscopy (EM). As shown in Figure 2B, in the control groups (miR-CL and miR-362), compact and well-organized desmosomes were observed with typical opposing electron dense plaques anchoring on the cell membranes. Multiple desmosomes were also found in some areas at the boundary of the cardiomyocytes. In contrast, most miR-21 mimic transfected cells demonstrated no apparent desmosomes despite multiple cell-cell contacts. In a few miR-21 mimic transfected cells, desmosome-like structures with much thinner and shorter electron dense plaques were identified. For the 100 cells analyzed in each group, 62 and 55 desmosomes were observed in miR-CL and miR-362 group, respectively; while only 17 desmosome-like structures were found in the miR-21 mimic transfected group. To confirm these findings, the distribution of γ-catenin was analyzed by immunofluorescence staining. Loss of γ-catenin at the cell-cell connection sites accompanied with increased intracellular localization was observed in miR-21 transfected cells compared with miR-CL and miR-362 groups (Figure S5A in Text S1). These data indicate that increased miR-21 level downregulates desmin expression and damages desmosome structures in cardiomyocytes.

**Figure 3:**
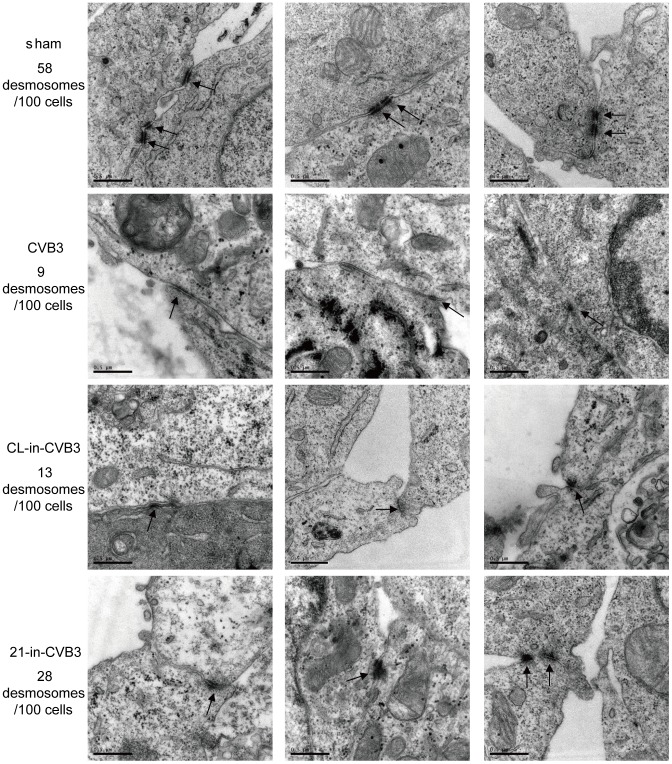
21-in rescues desmosome structures during CVB3 infection. HL-1 cells were transfected and infected as indicated. “Sham” and “CVB3” are plain HL-1 cells infected with PBS or CVB3 at 10 MOI for 24 h, respectively. “CL-in-CVB3” and “21-in-CVB3” are HL-1 cells transfected with CL-in or 21-in and then infected with CVB3 at 10 MOI for 24 h. Desmosomes were analyzed by EM and indicated with black arrow. For each sample, 100 cells were analyzed and the numbers of desmosomes observed were indicated. Magnification: 37000×. Bar: 0.5 µm.

As shown in Figure 2A, even in control groups that were not transfected with miR-21, CVB3 infection can also induce desmin downregulation. We then investigated the influence of CVB3 infection on desmosomes. EM data showed that CVB3 infection also resulted in fewer, thinner and shorter desmosomes in comparison to the sham controls (Figure 3). More importantly, inhibition of cellular miR-21 by 21-in partially rescued the desmosomes by maintaining the proper structures of some desmosomes and increasing the desmosome numbers. These observations were validated by detection of γ-catenin using immunofluorescence staining. Infection of CVB3 led to loss of γ-catenin in the cell-cell contact sites while 21-in partially alleviated such reduction (Figure S5B in Text S1). Together, our data suggest that miR-21 induced by CVB3 triggers desmosome destruction in the infected cardiomyocytes.

### miR-21 Promotes Desmin Degradation through the Ubiquitin-Proteasome Pathway

**Figure 4:**
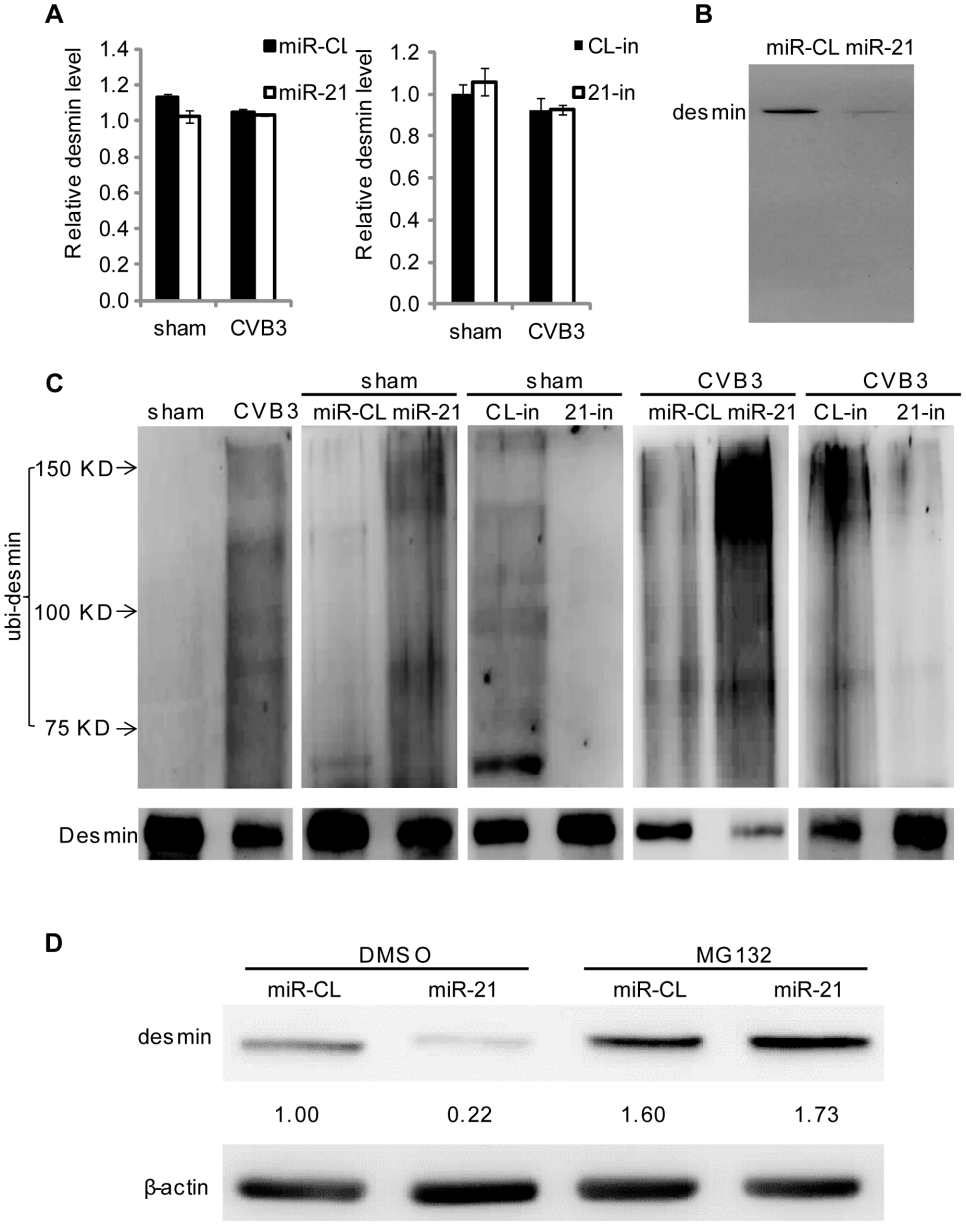
miR-21 promotes desmin degradation through the ubiquitin-proteasome pathway. (**A**) Neither miR-21 transfection nor CVB3 infection affects desmin transcription. HL-1 cells were transfected and infected as indicated. Total RNAs were isolated and subjected to q-RT-PCR detection of desmin mRNAs. Data was normalized to GAPDH. (**B**) miR-21 does not induce desmin cleavage. HL-1 cells were transfected as indicated. Desmin was analyzed by WB. (**C**) miR-21 enhances desmin protein ubiquitination during CVB3 infection. HL-1 cells were transfected and infected as indicated. Desmin was pulled down by immunoprecipitation and analyzed by WB to detect ubiquitin. (**D**) Proteasome inhibitor blocks the effect of miR-21 on desmin degradation. HL-1 cells were transfected as indicated. DMSO or MG132 was added after transfection. Desmin levels were detected by WB.

To reveal the mechanism by which miR-21 suppresses desmin expression, we first evaluated it at the transcription level by q-RT-PCR. The results proved that neither CVB3 infection nor miR-21 transfection/inhibition could cause alterations in desmin mRNA levels (Figure 4A). This suggests that the change of desmin expression occurs at the post-transcriptional level. We then performed bioinformatic prediction using the miRWalk software [29] to search for potential miR-21 targeting sites within desmin mRNA but no site was found in the 5′ untranslated region (5′UTR), coding region or the 3′UTR, indicating no direct targeting effect of miR-21 on desmin translation. Previous studies reported that desmin undergoes cleavage by protease such as cystein protease and caspase [30], [31]. However, our western blot (WB) analysis showed no cleavage products despite a robust decrease in desmin protein levels in the miR-21 mimic transfected samples (Figure 4B). Recently, it was reported that desmin is susceptible to degradation via ubiquitin-proteasome pathway [32]. We thus further determined whether miR-21 regulates desmin ubiquitination. To this end, desmin was pulled down by immunoprecipitation and the ubiquitinated desmin (ubi-desmin) was detected by an anti-ubiquitin antibody. We found that CVB3 infection or transfection of miR-21 mimics induced poly-ubiquitination of desmin while 21-in suppressed such process (Figure 4C). In CVB3 infected cells, transfection of miR-21 mimics further intensified the ubiquitination of desmin while 21-in alleviated this process. To further confirm the involvement of ubiquitin-proteasome pathway in miR-21 mediated desmin degradation, we applied proteasome inhibitor MG132 to block this pathway. As shown in Figure 4D, compared with DMSO control, MG132 eliminated the effect of miR-21 on desmin downregulation. These data indicate that miR-21 promotes desmin degradation through the ubiquitin-proteasome pathway.

### miR-21 Specifically Targets YOD1

**Figure 5:**
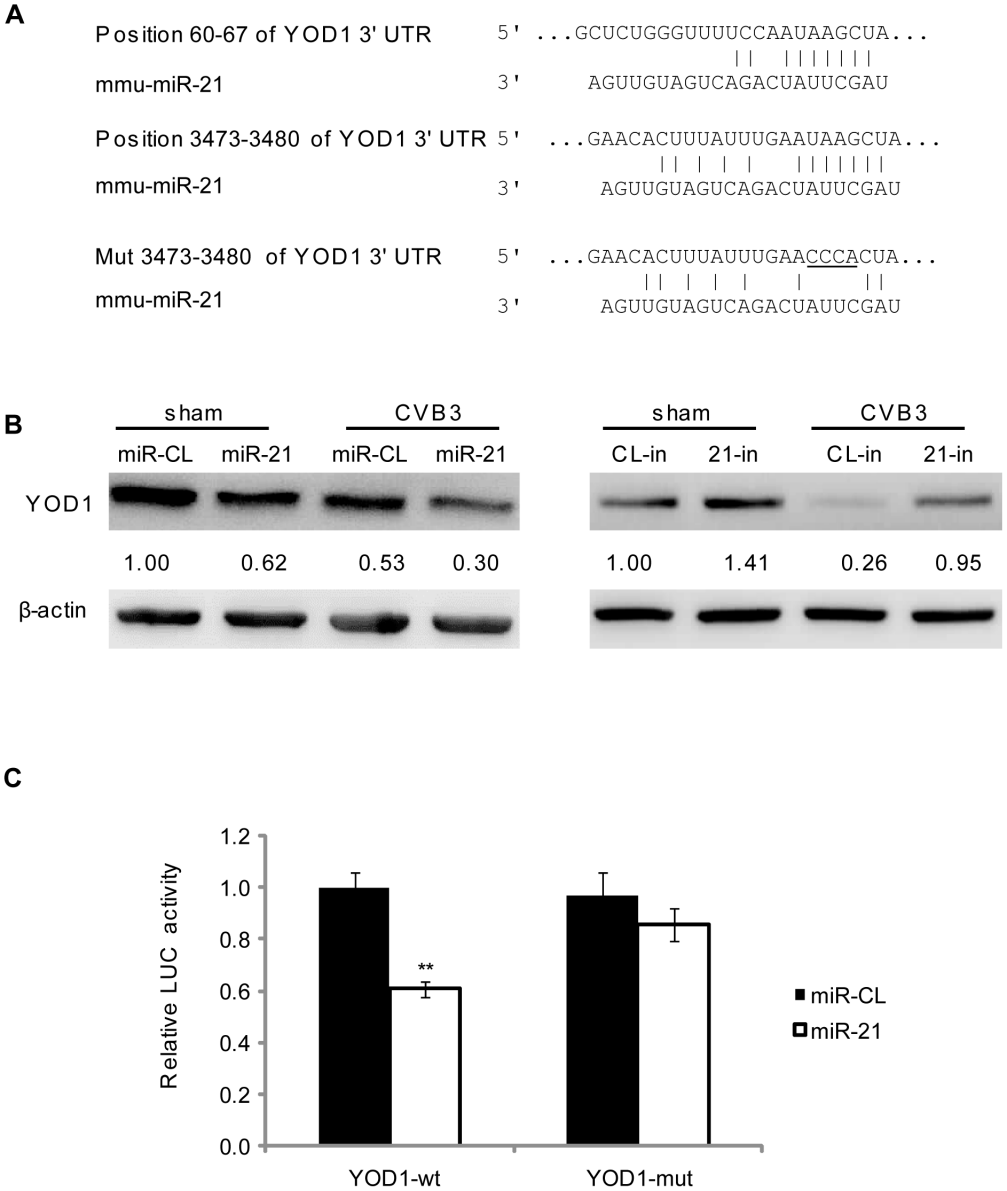
miR-21 targets YOD1. (**A**) Wt and mut miR-21 targets within the 3′UTR of murine YOD1 mRNA. The target sites were predicted by using TargetScan program. Mutated sites were designed for luciferase assay controls. (**B**) miR-21 suppressed YOD1 expression. HL-1 cells were transfected and infected as indicated. YOD1 expression levels were measured by WB. (**C**) Luciferase assay to validate miR-21 targeting effect on YOD1 translation. HL-1 cells were co-transfected with miRNA mimics and luciferase reporters harboring wt or mut YOD1 3′UTR fragments. Dual luciferase assays were conducted to compare the relative luciferase activities (Firefly/Renilla) among different groups.

To understand the mechanism by which miR-21 induces desmin ubiquitination, we performed a bioinformatic search for the potential target genes of miR-21 using the TargetScan software [28]. Among the top 10 predicted targets, YOD1 is a known mediator of the ubiquitin-proteasome pathway (Table S1 in Text S1). Two conserved targeting sites were identified on the 3′UTR of YOD1 mRNA (Figure 5A). YOD1 is a deubiquitinating enzyme that removes ubiquitin residues from the ubiquitinated proteins, facilitating the dislocation of mis-folded proteins from the endoplasmic reticulum (ER) for further degradation [33]. It is however not clear whether YOD1 regulates the degradation of normal cytosolic protein. To verify whether YOD1 is a true target of miR-21, we first measured the expression level of YOD1 in miR-21 mimic transfected cells without CVB3 infection. WB results showed that miR-21 mimic transfection led to a ∼40% (from 1.00 to 0.62) reduction in YOD1 levels compared with miR-CL. Similar reduction of YOD1 by miR-21 was observed in CVB3-infected samples (Figure 5B). We further used miR-21 inhibitors to knockdown the endogenous miR-21 and thus block the induction of miR-21 by CVB3 infection. A ∼40% (from 1.00 to 1.41) increase in YOD1 was observed in 21-in transfected samples compared with CL-in in sham infected samples. Importantly, 21-in transfection attenuated the dowregulation of YOD1 during CVB3 infection, indicating that CVB3-induced miR-21 is responsible for the reduction of YOD1 (Figure 5B). To validate the direct targeting effect of miR-21 on YOD1 3′UTR, we cloned one miR-21 targeting sites of YOD1 into a dual-luciferase reporter vector. We also constructed a mutated site by changing 4 base-pairs (bp) to disrupt the targeting effect of miR-21. Luciferase reporter assay showed that miR-21 caused ∼40% reduction in the luciferase activity of the reporter harboring the wide type (wt) site but not the mutant (mut) one (Figure 5C). These data demonstrate that miR-21 plays an essential role in downregulating YOD1 during CVB3 infection.

### Suppression of YOD1 Induces Desmin Degradation and Desmosome Disruption

**Figure 6:**
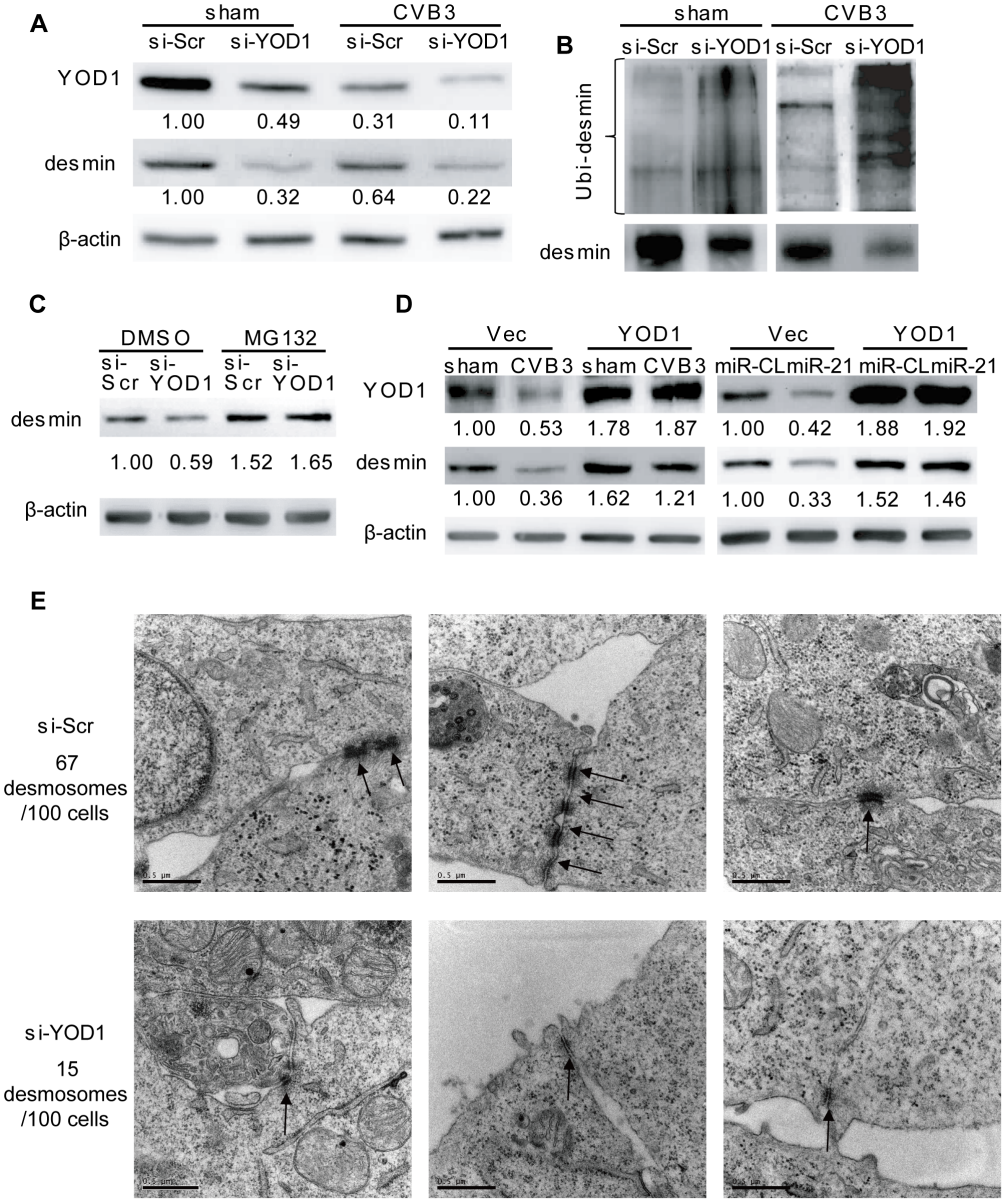
YOD1 regulates desmin degradation during CVB3 infection. (**A**) Silencing YOD1 expression downregulates desmin. HL-1 cells were transfected and infected as indicated. YOD1 and desmin proteins were detected by WB. (**B**) YOD1 siRNA enhances desmin ubiquitination. HL-1 cells were transfected and infected as indicated. Desmin was immunoprecipitated and analyzed by WB detection of ubiquitin. (**C**) Proteasome inhibitor blocks the YOD1 siRNA-mediated desmin degradation. HL-1 cells were transfected and treated with DMSO or MG132 as indicated. Proteins were extracted to detect the desmin levels. (**D**) Overexpression of YOD1 inhibits desmin degradation. HL-1 cells were divided into two groups: one group was transfected with empty vector or YOD1-expressing vector and then infected with CVB3; the other group was co-transfected with miRNA mimics and YOD1-expressing plasmid or empty vector. Desmin and YOD1 expression levels were evaluated by WB. (**E**) YOD1 siRNA disrupts desmosome structure. HL-1 cells were transfected with scrambled siRNAs or YOD1 siRNAs and the desmosome structures were analyzed by EM. Three representative views were listed for each sample and desmosomes were indicated with black arrows. For each sample, 100 cells were analyzed and the numbers of desmosomes observed were indicated. Magnification: 37000×. Bar: 0.5 µm.

zTo confirm that the YOD1 suppression by miR-21 leads to the degradation of desmin during CVB3 infection, we utilized small interference RNA (siRNA) to knockdown endogenous YOD1, which mimicked the effect of miR-21 on YOD1 expression. As shown in Figure 6A, compared with the scrambled control siRNA (si-Scr), YOD1 siRNA (si-YOD1) successfully suppressed YOD1 expression in both sham- and CVB3-infected cells, and this suppression correlated well with each corresponding decrease of desmin protein. Particularly, in CVB3-infected samples, desmin was further downregulated compared to the sham-infected control, indicating the role of YOD1 in desmin degradation. We also found more ubiquitinated desmin proteins in si-YOD1 transfected cells than in the control group in both sham- and CVB3-infected samples (Figure 6B). Application of MG132 blocked the degradation of desmin, which is consistent with the miR-21 mimic transfection results (Figure 6C). To further confirm that YOD1 inhibition and desmin downregulation is mediated by miR-21, we transfected cells with miR-362 as an additional non-specific control, which showed no effect on the expression levels of γ-catenin, desmin, YOD1 and ubiquitinated desmin (Figure S6A–B in Text S1).

The above finding was further verified by ectopic expression of YOD1 in CVB3 infected or miR-21 transfected cells. The results showed that similar levels of desmin expression were found in sham- and CVB3-infected cells in the presence of a YOD1 expression plasmid while the empty vector failed to rescue the loss of desmin (Figure 6D). The overexpression of YOD1 almost eliminated the effect of miR-21 on desmin degradation as evidenced by the equivalent levels of desmin in miR-CL and miR-21 transfected cells in the presence of a YOD1 expression plasmid. These data indicate that overexpression of YOD1 could counteract the effect of miR-21 on desmin degradation and that YOD1 is responsible for the miR-21 mediated degradation of desmin during CVB3 infection.

Further, we examined the effect of YOD1 siRNA on desmosome structure in cardiomyocytes. EM data showed that silencing YOD1 resulted in substantial reduction of desmosome number and weakening of desmosome structure compared with the controls. These are evidenced by the thinner and shorter electron dense plaques and fewer desmosome-like structure (15/100 cells) in YOD1 siRNA-transfected cells than in the control group (67/100 cells) (Figure 6E). This observation was supported by immunofluorescence staining of γ-catenin, showing loss of γ-catenin at the cell-cell contact area due to the knocking down of YOD1 (Figure S6C in Text S1).

**Figure 7:**
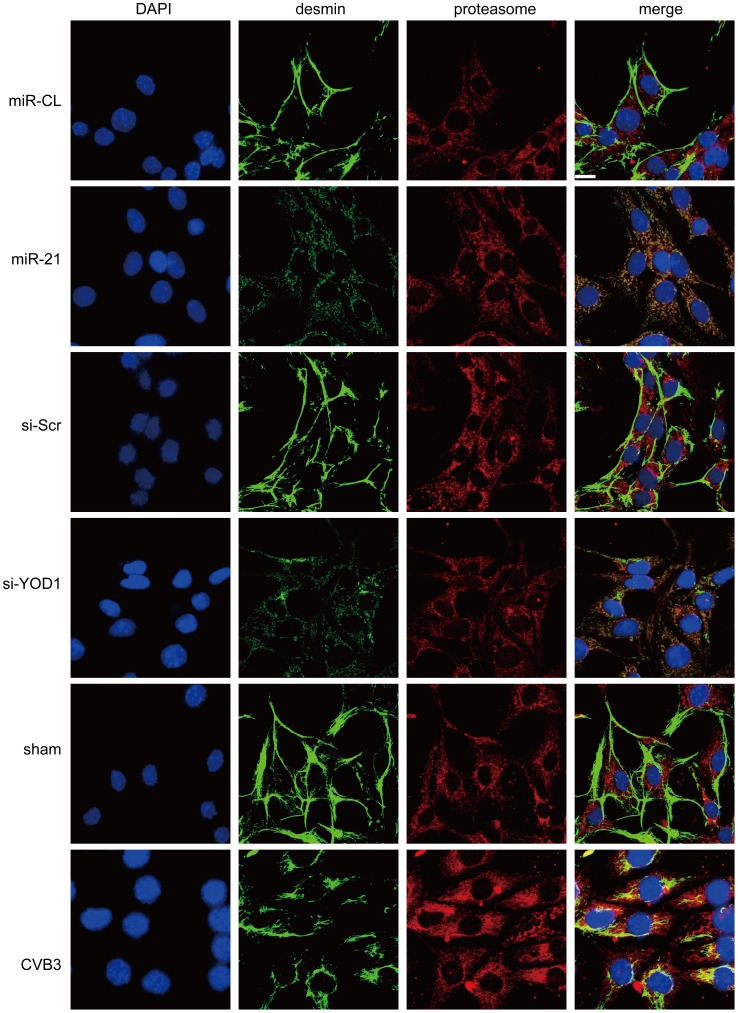
miR-21, YOD1 siRNA or CVB3 infection induces co-localization of desmin proteins and proteasomes. HL-1 cells were transfected as indicated or infected with CVB3 at 10 MOI for 24 h. Cells were subjected to immunofluorescence detection of desmin and proteasome. Nuclei were stained by DAPI (blue). Images were captured by confocal microscopy. Bar: 20 µm.

We then investigated the effect of miR-21 and YOD1 siRNA on the distribution of desmin protein in cardiomyocytes. Immunofluorescence staining data showed that in control groups (miR-CL, si-Scr and sham), desmin was mainly localized along the cell borders where the cells contact with each other. On the contrary, transfection of miR-21 mimics, knocking down of YOD1 or infection of CVB3 induced more cytoplasmic distribution of desmin than the controls (Figure 7). More importantly, we found that the redistribution of desmin was accompanied by the increased (2–3 folds) co-localization of desmin and proteasomes, which was verified by the Pearson's Correlation analysis (Figure S7 in Text S1). These results support our findings that miR-21-induced suppression of YOD1 promotes desmin degradation through the ubiquitin-proteasome pathway during CVB3 infection, which contributes to the damage of desmosomes.

**Figure 8:**
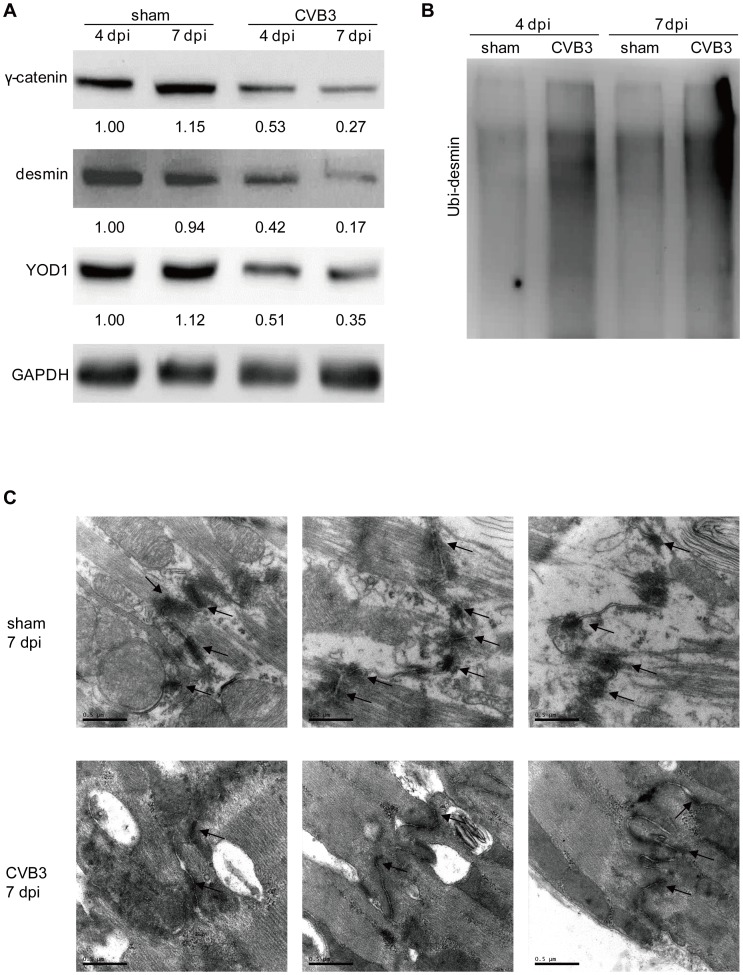
CVB3 infection disrupts desmosomes *in vivo*. 4-week old A/J mice were infected with CVB3 for 4 or 7 days. Heart proteins were harvested for detection of γ-catenin, desmin, and YOD1 (**A**) or for desmin ubiquitination assay (**B**). The intensities of the bands were measured by using ImageJ and the signal ratios were listed below. Cardiac desmosome structures were analyzed by EM (**C**). Three representative views were listed for each sample and desmosomes were indicated with black arrows. Magnification: 37000×. Bar: 0.5 µm.

To test whether CVB3 infection *in vivo* regulates the YOD1-desmin cascade, we detected the expression levels of desmosome components and desmosome structures in the CVB3 infected mouse heart. Compared with the sham control, CVB3 infection downregulated the expression levels of γ-catenin, YOD1 and desmin (Figure 8A), while increased the desmin ubiquitination level (Figure 8B). More importantly, EM analysis demonstrated that CVB3 infection triggered the loss of normal dark and thick desmosomes between cardiomyocytes and resulted in shorter, thinner and smaller desmosomes (Figure 8C), similar to the *in vitro* results. These data imply that CVB3 interrupts cardiomyocyte connections by modulating the YOD1-desmin cascade.

### Lysine 48-Linked Polyubiquitination Mediates Desmin Degradation

**Figure 9:**
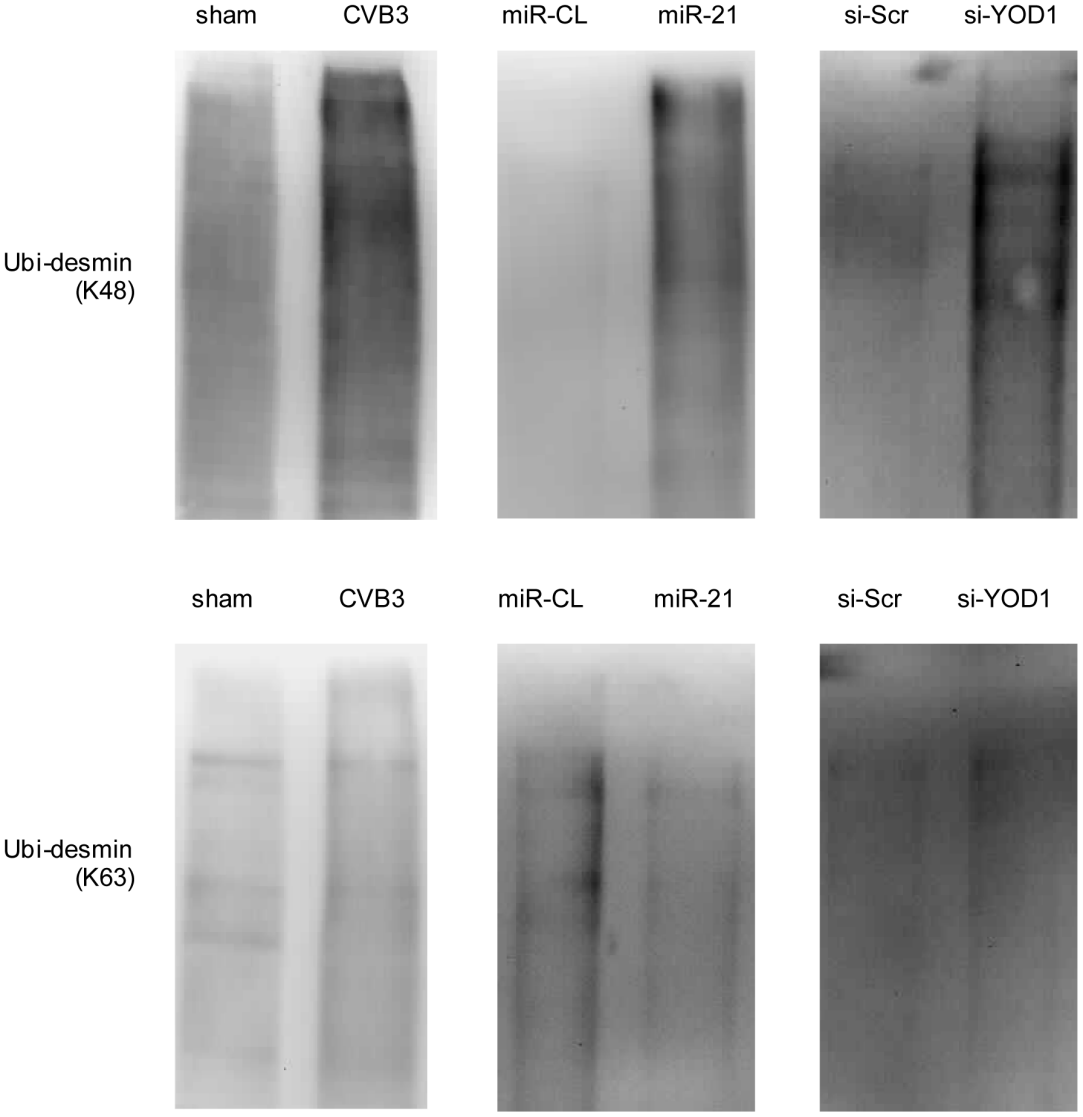
miR-21 mediates K48-linked polyubiquitination of desmin during CVB3 infection. HL-1 cells were transfected as indicated or infected with CVB3 at 10 MOI for 24 h. Cellular proteins were collected and pulled down by an anti-desmin antibody and subjected to WB detection of K48- or K63-linked polyubiquitin chains.

**Figure 10:**
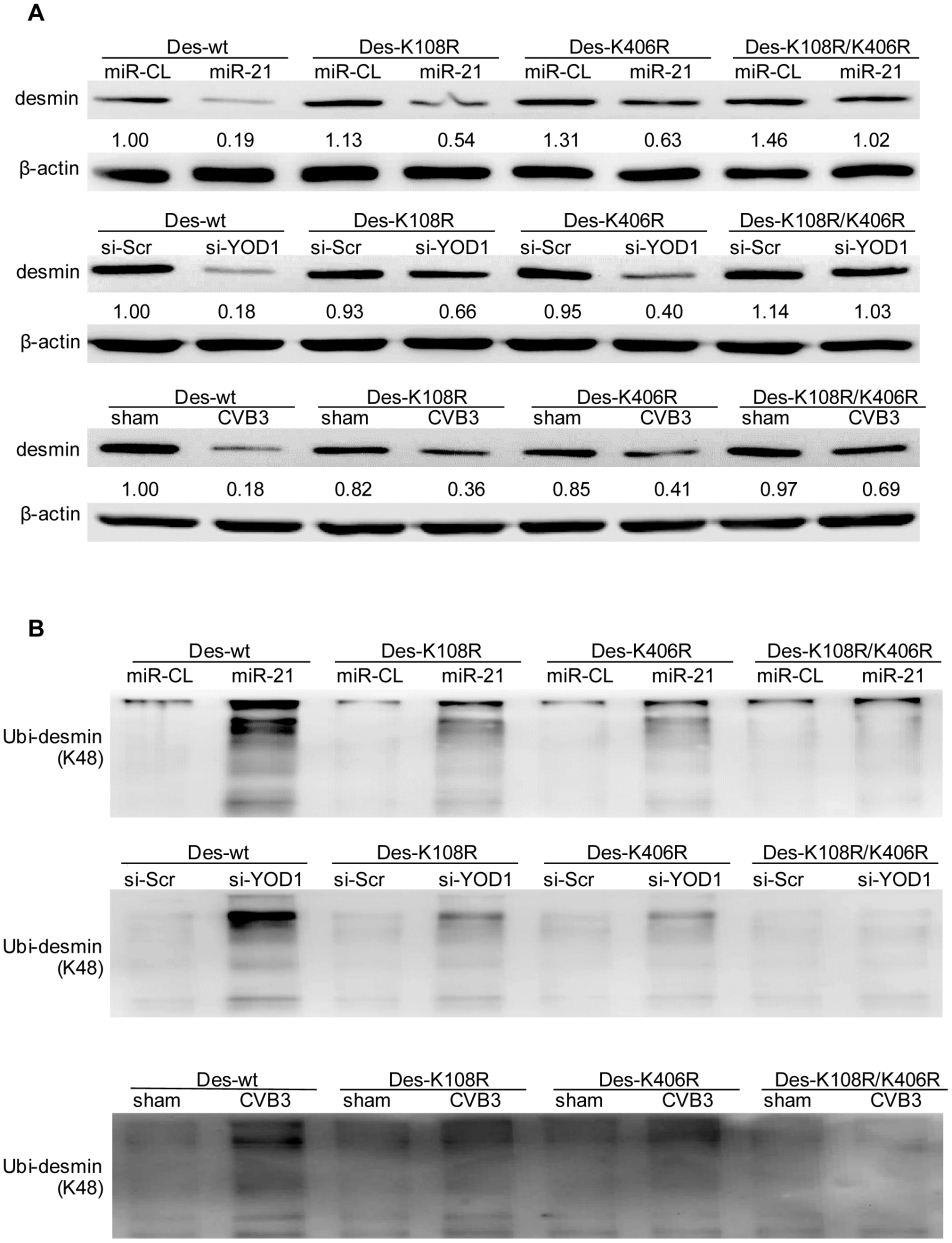
K108 and K406 are essential sites for desmin ubiquitination and degradation. HL-1 cells were co-transfected with miRNA mimics, siRNAs and desmin plasmids (wild type or mutants) or infected with CVB3 as indicated. Cellular proteins were collected for detection of desmin expression level using WB. The intensities of the bands were measured by using ImageJ and the signal ratios were listed below (**A**). The proteins were also pulled down by an anti-desmin antibody and subjected to WB detection of K48-linked polyubiquitin chains (**B**).

The linkage between polyubiquitin chains and the target proteins can be mediated by lysine 48 (K48) or 63 (K63) which affects the fate of the ubiquitinated proteins [34]. We tested whether the desmin ubiquitination is linked by K48 or K63. In cells treated with miR-21, YOD1 siRNA or infected with CVB3, the K48 linked rather than the K63-linked ubiquitination was increased compared with the control groups (Figure 9). Previous studies suggested that eleven lysine residues of the mouse desmin protein are involved in its ubiquitination [35]. Among them, K108 and K406 are conserved in the human desmin protein for ubiquitination [36], [37]. We thus introduced point mutations into these two sites and studied the effect of these mutations on desmin ubiquitination and degradation. As shown in Figure 10, single mutation at K108 or K406 (K108R or K406R) attenuated the ubiquitination and downregulation of desmin induced by miR-21, YOD1 siRNA or CVB3 infection compared with the wt desmin. Such effect was enhanced by introducing a double mutation at both K108 and K406 (K108R/K406R). These above data indicate that K48-linked polyubiquitination of desmin at K108 and K406 contributes to the miR-21 mediated desmin degradation during CVB3 infection.

### miR-21 Interrupts Fascia Adherens in Human Cardiomyocytes by Targeting VCL

**Figure 11:**
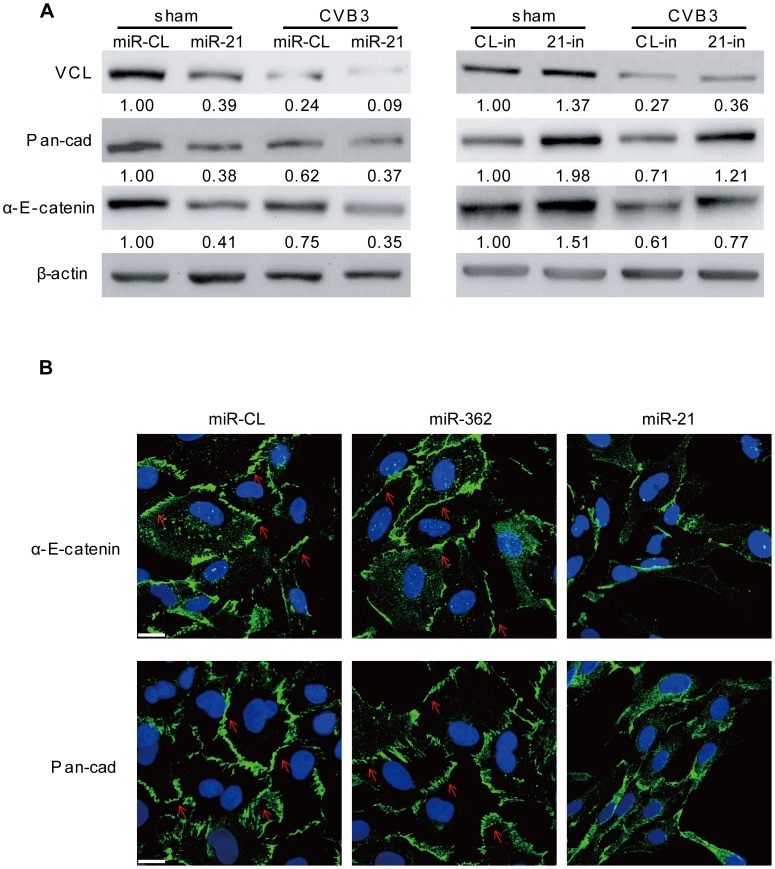
miR-21 interrupts fascia adherens during CVB3 infection. HL-1 cells were transfected and infected as indicated. VCL and other indicated proteins involved in fascia adherens were detected by WB (**A**). Distribution of pan-cadherin and α-E-catenin were investigated by immunofluorescence staining and confocal microscopy (**B**). Blue represents the nuclei stained by DAPI and green shows the expression of pan-cadherin or α-E-catenin as indicated. Red arrows label the localization of pan-cadherin or α-E-catenin along the cell borders where cardiomyocytes contact each other.

Among the top 10 miR-21 targets, another gene, VCL, is also closely related to the regulation of cell-cell connections and cardiac function. VCL is an essential component of fascia adherens that maintains cardiac structures [23]. Through bioinformatic prediction, we found one conserved targeting site of miR-21 in the VCL mRNA of both human and mice; while human VCL contains an additional miR-21 site (Figure S8A in Text S1). We first performed luciferase assay to evaluate the targeting effect of miR-21 on the conserved site. The results showed that miR-21 inhibited the luciferase activity of the reporter harboring the wt site but not the mutant one, indicating the specific recognition of this site by miR-21 (Figure S8B in Text S1). We then measured the expression of VCL in different human and mouse cell lines that transfected with miR-21 mimics. Interestingly, in all three human cell lines, immortalized human cardiomyocytes, HeLa cells and HEK 293T cells, miR-21 significantly inhibited VCL expression (Figure S8C in Text S1). In contrast, in the two mouse cell lines tested, NIH 3T3 and HL-1 cells, despite the successful transfection of miR-21 mimics as evidenced by q-RT-PCR results (Figure S3B in Text S1), only minimal inhibitory effect of miR-21 on VCL expression was observed (Figure S8C in Text S1). This indicates that the immortalized human cardiomyocytes would be a better cell line to study the role of miR-21 in fascia adherens. We thus transfected these cells either with miR-21 mimics to increase miR-21 level or with 21-in to suppress CVB3-induced expression of miR-21. The two cellular proteins (i.e., pan-cadherin and α-E-catenin) associated with fascia adherens were then detected by WB. The results showed that miR-21 silenced VCL expression, leading to downregulation of pan-cadherin and α-E-catenin compared with miR-CL and miR-362 (Figure 11A, S9A in Text S1). However, application of 21-in produced opposite results (Figure 11A). Knocking down of VCL using siRNAs showed similar effect as that of miR-21 on the inhibition of pan-cadherin and α-E-catenin (Figure S9B in Text S1). We further analyzed the fascia adherens by investigating the distribution of pan-cadherin and α-E-catenin by using the immunofluorescence staining method. In control cells (miR-CL, miR-362 and si-Scr), both signals were well aligned with the cell-cell connection border lines. However, when transfected with miR-21 mimics (Figure 11B) or VCL siRNAs (Figure S9C in Text S1), pan-cadherin and α-E-catenin were disorganized with the loss of clear cell contact sites. In addition, the irregular distribution of pan-cadherin and α-E-catenin was also observed in CVB3 infected cells, while inhibition of miR-21 could partially inhibit such phenomenon, particularly the localization of α-E-catenin (Figure S10 in Text S1). These data imply that miR-21 targets VCL and interrupts fascia adherens during CVB3 infection.

## Discussion

miRNAs are among the pivotal regulators of virus-host interactions. Several studies have reported the role of host miRNAs in regulating the replication of CVB3 and the activation of inflammatory process [18]–[20], [22]. However, the research on the roles of miRNAs in cardiomyocyte pathology during the occurrence of viral myocarditis, particularly the regulation of cell-cell connections which are fundamental to cardiac structures and functions, is still missing. This study is the first to reveal the role of miR-21 in modulating ICDs in the cardiomyocytes during CVB3 infection. We identified two new targets, YOD1 and VCL, of miR-21. Suppression of YOD1 by miR-21 promoted desmin degradation and desmosome disorganization. Targeting VCL by miR-21 directly triggered the reduction and disorientation of fascia adherens components including pan-cadherin and α-E-catenin. These findings provide a new perspective to understand the role of miRNAs in viral myocarditis.

The differential expression of miR-21 caused by CVB3 infection is controversial. Both up- and down-regulation of miR-21 expression during CVB3 infection have been reported [15], [19], [22]. In our study, we found by microarray analysis that CVB3 infection induced miR-21 upregulation. We also further confirmed the results by q-RT-PCR using both the *in vivo* and *in vitro* models at different time points post infection. It is worth noting that different studies used different mouse strains including C3H, BALB/c and A/J and also employed distinct endogenous controls such as GAPDH and U6. These may partially explain the inconsistency of the data. Considering that three independent groups including ours found that miR-21 is increased and that the transcriptional factors controlling miR-21 expression, such as activation protein 1 (AP-1) [38], STAT3 [39] and p38 [40], are all activated by CVB3 infection [20], [41], [42], we would argue that miR-21 is indeed upregulated by CVB3 infection in the heart. The two major cell types in adult murine hearts are cardiomyocytes (∼56%) and cardiac fibroblasts (27%) [43]. During CVB3 infection, about 30% of the cardiomyocytes are infected [44]. In our study, both mice and human cardiomyocytes showed 5–10 fold increase in miR-21 expression levels during CVB3 infection, suggesting the contribution of cardiomyocytes to overall miR-21 upregulation in CVB3-infected hearts. It has been reported that miR-21 is a central regulator for fibrosis [45], indicating that cardiac fibroblasts may also associate with the miR-21 increase during CVB3 infection though further validation is still needed. The infiltrated CD45+ inflammatory cells account for 20–30% of the total cells in the infected heart during viral myocarditis [46], [47]. It has recently been reported that miR-21 upregulation is a general feature of T-cell activation [48], implying a likely involvement of immune cells in miR-21 induction in viral myocarditis. In addition, we showed that UV-irradiated CVB3 failed to induce miR-21, suggesting that active replication of the virus is required for such induction.

ICDs are composed of three major sub-units, desmosomes, fascia adherens and gap junctions [23]. They are the anchoring pillar to stabilize the cardiac structures and the bridging cable to transmit signals among cardiomyocytes. It is known that deficiency in ICDs causes both constitutional and functional damage to the myocardium. CVB3 infection has been found to affect endothelial tight junction [49] but its pathologic effect on cardiomyocyte ICDs is not clear. miR-21 has been identified as one of the most important miRNAs involved in heart diseases, particularly in cardiac hypertrophy [7], [11]. However, its role in modulating ICD organization has been very limitedly investigated. Desmosomes are symmetrical protein complexes connecting adjacent cardiomyocytes. They are tightly linked by desmin, an intermediate filament facilitating the anchoring of desomosomal plaques [50]. Mutation or knockout of desmin frequently causes dilated cardiomyopathy [51], [52], a heart disease that often develops in the end stage of viral myocarditis [27]. Knockout of desmin also leads to fibrosis and ischemia injury in the heart with the loss of myocardium strength and integrity [53]. Cardiomyocytes lacking desmin show disorganized myofibrils which are separated from ICDs, resulting in a reduction in desmosome numbers [54]. In this study, we found that desmin levels were reduced by miR-21 mimic transfection and CVB3 infection. Silencing miR-21 could rescue the desmin levels during CVB3 infection, suggesting the regulatory role of miR-21 in desmin levels in CVB3-infected cardiomyocytes. Our results showed that miR-21 could not suppress desmin transcription. Further, we failed to observe any protease-mediated cleavage of desmin though it might be due to that the antibody we used could not recognize the cleavage products. Recent studies reported the loss of desmin due to ubiquitination by Trim32 during muscle atrophy [32], indicating the important role of ubiquitin-proteasome system in modulating desmin levels. We found that CVB3 infection and/or miR-21 mimic transfection enhanced desmin ubiquitination while 21-in produced an opposite effect. Application of proteasome inhibitor suppressed the miR-21-mediated desmin degradation. These data suggest that CVB3-induced miR-21 expression causes desmin degradation through the ubiquitin-proteasome pathway. Importantly, we further demonstrated that the suppressed desmin levels eventually led to quantity reduction and structure disorganization of desmosomes. Inhibition of miR-21 during CVB3 infection hindered the desmosome loss and destruction.

In search for the underlying mechanism by which miR-21 promotes desmin degradation, we identified YOD1 as a novel target of miR-21. YOD1 is a deubiquitinating enzyme (DUB) in the ovarian tumor (OTU) family that removes ubiquitin residues from poly-ubiquitinated proteins [14]. It is involved in the degradation of misfolded proteins in ER. DUBs in OTU family have been found to be capable of stabilizing some cytosolic proteins. OTUB1 stabilizes c-IAP1 by counteracting its ubiquitination process [55]. It also enhances the stability of p53 by suppressing its ubiquitination [56]. The role of YOD1 in protein stabilization is unknown. By using siRNA to knock down YOD1, enhanced desmin ubiquitination and degradation was observed. Treatment of proteasome inhibitor blocked the effect of YOD1 siRNAs on desmin suppression, suggesting that YOD1 is essential to stabilize desmin. YOD1 siRNAs also caused disruption of desmosomes. These results are in line with the role of miR-21 in promoting desmin degradation. Furthermore, overexpression of YOD1 attenuated the effect of CVB3 infection and/or miR-21 transfection on desmin degradation. Interestingly, both miR-21 and YOD1 siRNAs induced the re-distribution of desmin, resulting in increased co-localization of desmin and proteasomes. Additionally, our *in vivo* data also showed that CVB3 infection led to downregulation of YOD1 and ubiquitin-proteasome mediated desmin degradation, which causes subsequent desmosome destruction in the mouse heart. These findings suggest that YOD1 possesses a similar function to OTUB1 in stabilizing certain cellular proteins and that suppression of YOD1 expression during CVB3 infection is the cause of desmosome damage.

Ubiquitin contains seven lysine residues (K6, K11, K27, K29, K33, K48 and K63) and a N-terminal methionine (M1) which potentially contribute to the linkage of polyubiquitin chain to targeted proteins [57]. The canonical K48-linked polyubiquitin chains are frequently associated with the proteasome-mediated degradation of targeted substrates while the K63-linked ones are often involved in protein trafficking, DNA repair and inflammation processes [58]. In this study, we found that CVB3, miR-21 or YOD1 siRNA stimulated K48- rather than K63-linked polyubiquitination, supporting that these treatments promote desmin degradation via the proteasome system. Further, we identified K108 and K406 located in the desmin protein as important sites for desmin ubiquitination and degradation. Mutation of these two sites inhibited desmin downregulation during CVB3 infection. Indeed, the introduction of mutation did not fully rescue the desmin level. There are several possible explanations. First, the detection of total desmin in the samples did not exclude endogenous wild type desmin which still can be regulated by miR-21 and CVB3. This can be further tested with tagged desmin. Second, there may be other residues in desmin involved in its ubiquitination and degradation. Third, ubiquitination may not be the only mechanism for desmin downregulation, particularly during CVB3 infection which induces shut-off of host protein translation [59]. Further investigations may elucidate this mechanism.

Another major component of ICD is fascia adherens. VCL is one of the major components of fascia adherens. Cardiomyocyte-specific excision of VCL results in dilated cardiomyopathy and sudden death in young mice [60], both of which are typical consequences of CVB3-induced heart failure. We demonstrated that suppression of VCL by miR-21 led to the further inhibition of pan-cadherin and α-E-catenin, two cellular proteins associated with fascia adherens. This suppressed expression also caused disorientation of pan-cadherin and α-E-catenin in cell contact sites. The disappearance of distinct cell-cell connection sites supports the notion that VCL is essential for cell-cell contacts in cardiomyocytes. Inhibition of miR-21 attenuated the loss and redistribution of proteins in fascia adherens. Together, targeting of VCL by miR-21 yields a negative regulatory effect on fascia adherens during CVB3 infection.

In conclusion, we revealed a novel role of miR-21 on controlling the integrity of cardiomyocyte ICDs through targeting YOD1 and VCL. The disorganization of desmosomes and fascia adherens by miR-21 expression during CVB3 infection may contribute to the pathogenesis of viral myocarditis (Figure S11 in Text S1). Based on the understanding of the underlying mechanism of miR-21 function, new strategies can be developed for drug targeting to protect the integrity of the myocardium, which can be use to prevent or treat viral myocarditis.

## Materials and Methods

### Ethics Statement

This study was carried out in strict accordance with the recommendations in the Guide to the Care and Use of Experimental Animals – Canadian Council on Animal Care. Alls protocols were approved by the Animal Care Committee of Faculty of Medicine, University of British Columbia (protocol number: A11-0052).

### Animals, Cell Culture and Viral Infection

Male A/J mice (4-week old) were purchased from Jackson Laboratory. Mice were infected by intraperitoneally inoculation with 5×10^3^ plaque-forming unit (pfu) of CVB3 or sham-infected with phosphate buffer saline (PBS) (Sigma). HeLa, NIH 3T3 and HEK 293T cells were cultured in Dulbecco's modified Eagle's medium (DMEM) (Lonza) with 10% fetal bovine serum (FBS) (Sigma). HL-1 cells were cultured in Claycomb medium (Sigma) with 10% FBS. Immortalized human cardiomyocytes were purchased from Applied Biological Materials and cultured in Prigrow I medium with 10% FBS. Viral infection of cells was conducted by incubation with viruses for 1 h in a serum free medium followed by switching to complete medium. Details are given in the Supporting Information.

### UV Irradiation of CVB3

One mL of CVB3 stock was aliquoted to a 2 mL tube and kept on ice. UV irradiation was conducted in a UV Stratalinker 1800 (Stratagene) for 30 min with the virus tube kept 5-cm from the UV bulb. The viruses were then tested by infection of HeLa cells and WB detection of the absence of VP-1 to confirm the successful irradiation.

### Plaque Assay

The supernatants from CVB3 infected samples were collected and serially diluted. The diluted supernatants were added onto HeLa cells in 6-well plates (8×10^5^ cells/well). Cells were washed with PBS after 1 h of incubation with the supernatants, supplemented with 0.75% soft agar in DMEM and 10% FBS, and incubated for 3 days. After incubation, cells were fixed with Carnoy's fixative for 30 min and stained with 1% crystal violet. The viral plaques were counted and the virus titers were calculated as the plaque forming units per mL (pfu/mL). All the assays were conducted at least three times.

### RNA Extraction, Microarray Analysis and q-RT-PCR

Mouse heart and cellular RNAs were extracted using the miRCURY RNA Isolation Kits (Exiqon) according to the manufacturer's instructions. Part of the mouse heart RNAs (4 dpi and 7 dpi) were submitted to Exiqon (Denmark) for miRNA microarray analysis as described previously [61]. The remaining heart RNAs and cellular RNAs were then reverse transcribed using a TaqMan MicroRNA Reverse Transcription Kit (Life Technologies). Mature miRNA levels were detected by the TaqMan MicroRNA Assay (Life Technologies) using relative quantitative methods as described previously [18]. U6 RNA was detected as the endogenous control for data normalization. For desmin q-RT-PCR, the cellular total RNAs were reverse transcribed by using the superscript III first-strand synthesis system (Life Technologies) and detected using the QuantiTect SYBR Green PCR master mix (Qiagen). GAPDH was detected as an endogenous control. Primers were listed in Table S2 in Text S1. All q-RT-PCR experiments were repeated in triplicates with the no-template as a control.

### Western Blot

Mouse heart tissues and cultured cells were lysed with the RIPA lysis buffer. Proteins were separated by sodium dodecyl sulfate polyacrylamide gel electrophoresis (SDS-PAGE), transferred to nitrocellulose membranes and identified by immunoassay. Protein levels were quantified by using ImageJ program. All the experiments were conducted at least three times. More details can be found in the Supporting Information.

### Immunofluorescence and Confocal Microscopy

Cells cultured on glass cover slips (Thermo Fisher) were washed with PBS and fixed with methanol/acetone (1∶1) for 20 min at −20°C. Cells were then washed with TBS twice and blocked with 2.5% bovine serum albumin (BSA) (Sigma) in TBS for 1 h at room temperature followed by incubation with primary antibodies diluted in blocking buffer overnight at 4°C. Cells were then washed with TBS five times (5 min/time) at room temperature. Secondary antibodies diluted in blocking buffer were then added into the samples and incubated for 1 h at room temperature. Samples were then washed with TBS five times at room temperature. The cover slips were stained with DAPI (DAKO) and mounted onto microscope glass slides (Thermo Fisher) with nail oil. Images were captured using a Leica AOBS SP2 confocal microscope (Leica, Allendale, NJ) and analyzed by using Volocity software. Details on the antibodies used are listed in the Supporting Information.

### Electronic Microscopy

Cultured cells or mouse heart sections were washed with 0.1M sodium cacodylate buffer and fixed in the primary fixing solution (2.5% glutaraldehyde (Polysciences) in 0.1 M sodium cacodylate buffer) for 1 h. Cells were washed with 0.1 M sodium cacodylate buffer for 3×10 min and fixed with the secondary fixation solution (1% osmium tetroxide (Polysciences) and 1% potassium ferrocyanide in 0.1 M sodium cacodylate buffer) for 1 h followed by 3×10 min washes with distilled water. Cells were then collected in 1.5 mL Eppendorf tubes and dehydrated by incubating with acetone (30%, 50%, 70% and 90% for 15 min each followed by 100% acetone (3×10 min)). The samples were infiltrated with a mixture of acetone-Eponate 12 resin (Epon) (Ted Pella) (1∶1) for 1.5 h and then with acetone-Epon (2∶1) for overnight. The samples were further infiltrated with 100% Epon for 6 h, embedded in 100% Epon in a flat embedding mould and cut into thin sections of 60 nm thickness using a UC6 Ultramicrotome and viewed under a Tecnai 12 transmission electron microscope (FEI Inc.) For cultured HL-1 cells, 100 cells were analyzed for each sample to calculate the desmosome number.

### Desmin Ubiquitination Assay

Desmin from the cultured cells was pulled down by desmin antibody (Abcam) using a Pierce Crosslink IP Kit (Thermo Scientific) following the manufacturer's instructions. The enriched desmin was then separated by 6% SDS-PAGE and immunoanalyzed by using an anti-ubiquitin antibody (Thermo Scientific), K48-linkage specific polyubiquitin antibody (Cell Signaling Technology) or K63-linkage specific polyubiquitin antibody (Cell Signaling Technology).

### H&E Staining

The hearts were collected and fixed in 10% formalin. Tissues were then embedded in paraffin and sectioned for standard hematoxylin and eosin (H&E) staining to evaluate cardiac inflammation and damage using the methods described previously [62].

### Transfection of miRNA Mimics, siRNAs and miRNA Inhibitors

miRNA mimics (Life Technologies), siRNAs (Dharmacon) and miRNA inhibitors (Life Technologies) were transfected into cells using the Lipofectamine RNAiMAX reagent (Life Technologies) according to the manufacturer's instructions. Cells were subjected to further infection or collected for analysis at 48 h post transfection. For more details, please refer to Supporting Information.

### Treatment of Proteasome Inhibitor

Cells were transfected with miRNA mimics or siRNAs for 6 h and treated with proteasome inhibitor MG132 (Santa Cruz) or equal volume of DMSO (Sigma) at 10 µM for 24 h. Cellular proteins were then collected at 48 h post transfection for detection of reduced protein degradation by WB analysis.

### Constructs and Dual Luciferase Assay

The wt and mut binding sites of miR-21 within the 3′UTR of YOD1 or VCL were synthesized by Integrated DNA Technologies. Oligomers were annealed and inserted into the pmirGLO Dual-Luciferase miRNA Target Expression Vector (Promega) according to the manufacturer's instructions. The oligonucleotides used for annealing are listed in Table S2 in Text S1. Dual luciferase assays were conducted using Dual-Luciferase Reporter Assay System (Promega). YOD1 expression plasmid and the corresponding empty vector were purchased from Origene and transfected at the concentration of 1 µg/well in 6-well plates using lipofectamine 2000 (Life Technologies). A plasmid overexpressing wt mouse desmin was purchased from Origene and used for generating desmin mutants through the mutagenesis service from Topgenetech Inc.. These mutants include two single mutations (K108R and K406R) and one double mutation (K108R/K406R). All the vectors were confirmed by sequencing. More details are provided in the Supporting Information.

### Statistical Analysis

All experiments were repeated at least three times. The Student's *t*-test was used for the paired comparison among the samples. A *p* value<0.05 (labeled with “*”) in two-tailed tests was considered as statistically significant. “**” was used for labeling differences with *p* value<0.01.

## Supporting Information

Text S1 Contains 11 figures, 2 tables and supplemental experimental procedures.
(PDF)
