# Supplementary material for: Coxsackievirus-Induced miR-21 Disrupts Cardiomyocyte Interactions via the Downregulation of Intercalated Disk Components
Source: PLoS Pathog. 2014 Apr 10;10(4):e1004070. doi: 10.1371/journal.ppat.1004070 (PMC3983067; doi:10.1371/journal.ppat.1004070)
Supplement: Text S1 — Contains 11 figures, 2 tables and supplemental experimental procedures. (PDF) [file ppat.1004070.s001.pdf]

Supporting Information for: Coxsackievirus-induced miR-21 disrupts cardiomyocyte interactions via the downregulation of intercalated disk components

Xin Ye[1], Huifang Mary Zhang[1], Ye Qiu[1], Paul J Hanson[1], Maged Gomaa Hemida[1], Wei Wei[2], Pamela A Hoodless[2], Fanny Chu[1] and Decheng Yang[1] *

1. Department of Pathology and Laboratory Medicine, University of British Columbia, The Centre for Heart Lung Innovation, St. Paul's Hospital, Vancouver, B.C., Canada

2. Terry Fox Laboratory, British Columbia Cancer Agency, Vancouver, B.C., Canada.

Inventory of Supplemental Information:

Figure S1. Confirmation of myocarditis occurrence and certain miRNA differential expression after CVB3 infection.
Figure S2. UV-irradiated CVB3 does not induce miR-21 expression.
Figure S3. miR-21 levels in different cell lines after transfection with either miRNA mimics or its inhibitors.
Figure S4. miR-21 does not affect CVB3 replication.
Figure S5. CVB3 induces miR-21 to affect γ-catenin distribution.
Figure S6. Knocking down of YOD1 affects γ-catenin distribution.
Figure S7. Pearson's Correlation analysis of co-localization of desmin proteins with proteasomes induced by miR-21, YOD1 siRNA or CVB3.
Figure S8. miR-21 targets VCL.
Figure S9. Knocking down of VCL interrupts fascia adherens.
Figure S10. Knocking down of miR-21 rescues fascia adherens during CVB3 infection.
Figure S11. A putative model of miR-21 regulation on ICD integrity during CVB3 infection.
Table S2. Oligomers used for cloning and q-RT-PCR
Supplemental Experimental Procedures

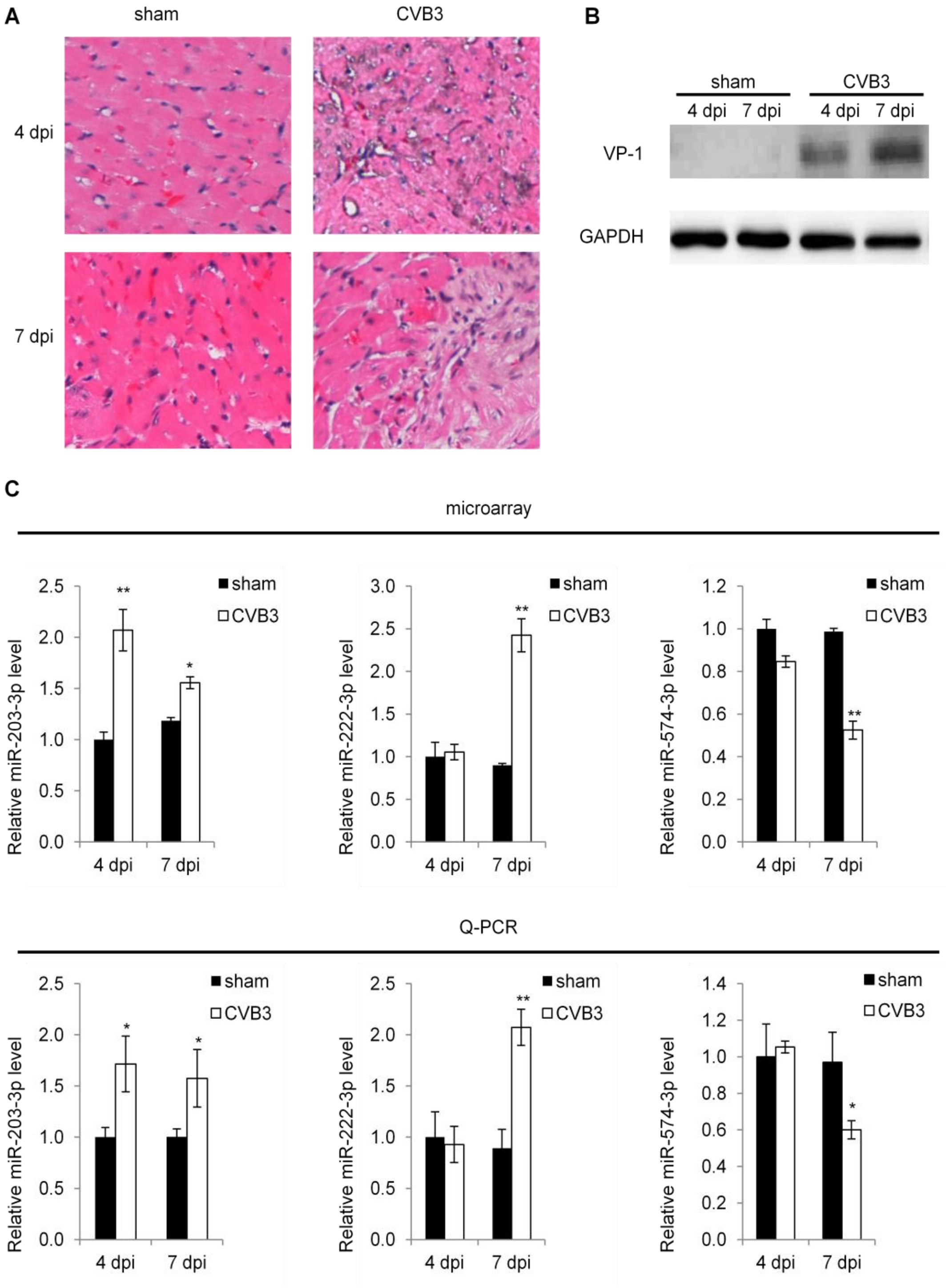

Figure S1. Confirmation of myocarditis occurrence and certain miRNA differential expression after CVB3 infection. (A) H&E staining of sham- or CVB3-infected mouse hearts. **(B)** VP-1 detection of CVB3 infected mouse hearts by WB. **(C)** Comparison of altered miRNA expression in CVB3 infected mouse hearts identified by microarray analysis and q-RT-PCR evaluation. "*" stands for $p < 0.05$ and "**" means $p < 0.01$. For microarray data, n=3 and for q-RT-PCR, n=5.

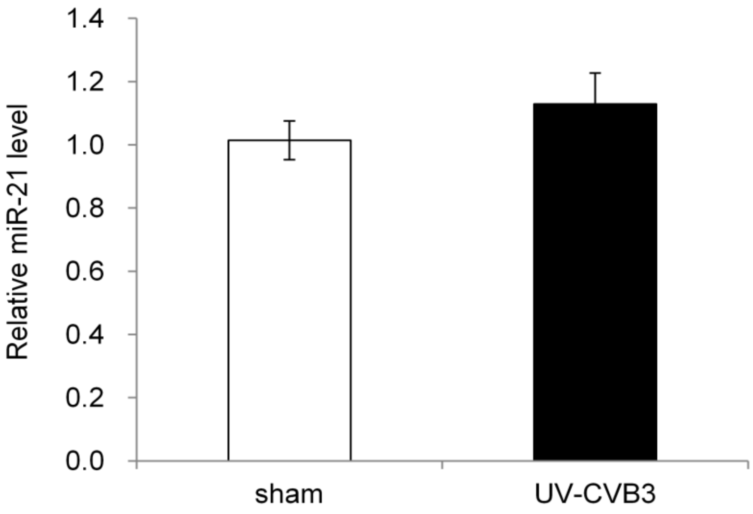

Figure S2. UV-irradiated CVB3 does not induce miR-21. HL-1 cells were infected with UV-irradiated CVB3 for 24 h. Cellular RNAs were harvested for q-RT-PCR detection of miR-21. $p > 0.05$, n=4.

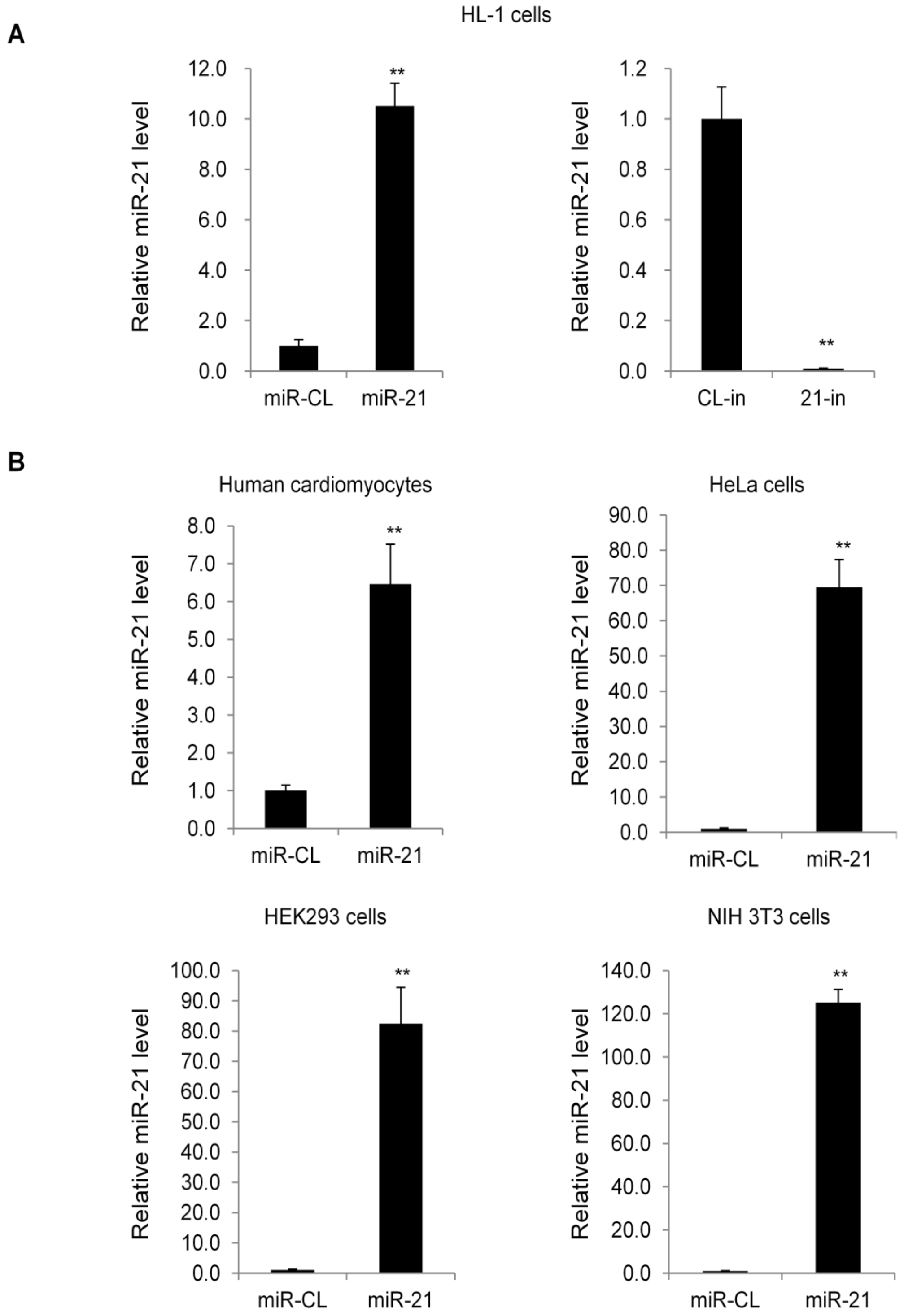

Figure S3. miR-21 levels in different cell lines after transfection with either miRNA mimics or its inhibitors. HL-1 cells **(A)** and other indicated cell lines **(B)** were transfected with miRNA mimics or inhibitors as indicated. miR-21 levels were measured by q-RT-PCR and normalized to U6 RNA. $p < 0.01$, n=4.

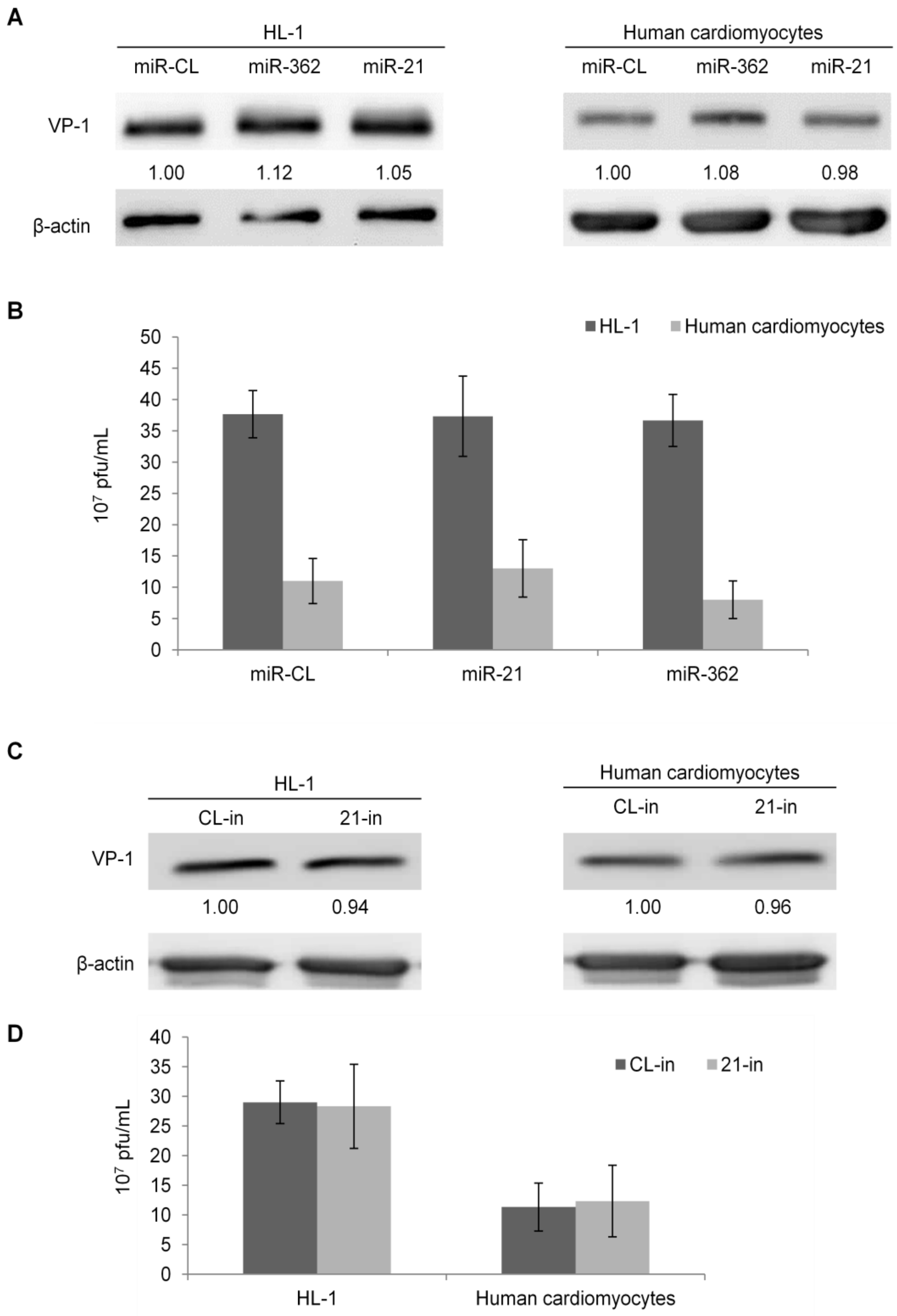

Figure S4. miR-21 does not affect CVB3 replication. HL-1 cells or immortalized human cardiomyocytes were transfected with miRNA mimics or inhibitors as indicated. Cells were then infected with CVB3 at 10 MOI (HL-1 cell) or 50 MOI (human cardiomyocytes) for 24 h. Cellular proteins were collected for detection of VP-1 levels **(A and C)**. Sample supernatants were harvested for viral plaque formation assay (**B and D**). $p > 0.05$, n=3.

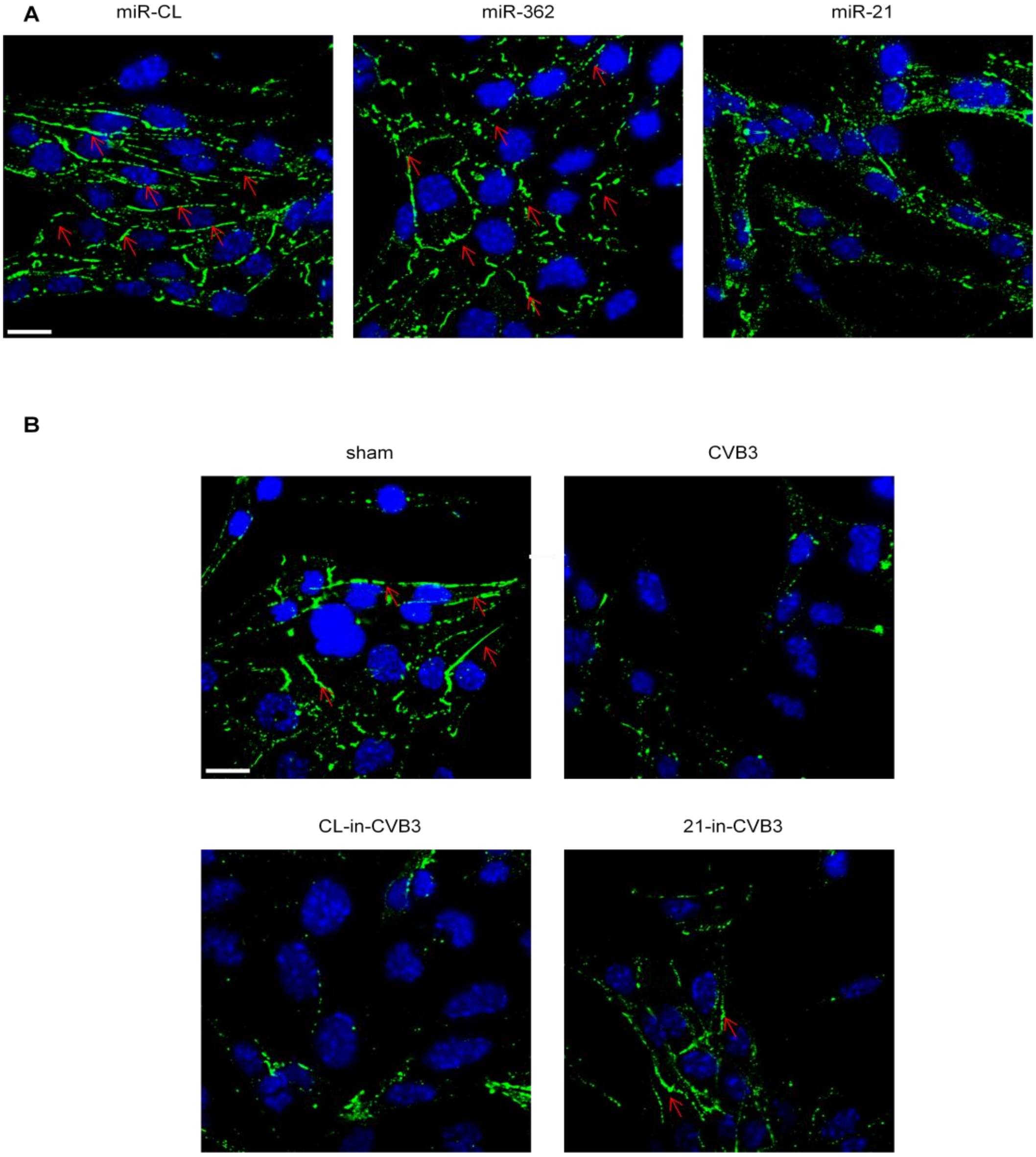

Figure S5. CVB3 induces miR-21 to affect γ-catenin distribution. HL-1 cells were transfected **(A)** or infected **(B)** as indicated. Cells were subjected to immunofluorescence detection of γ-catenin (green). Nuclei were stained with DAPI (blue). Red arrows label the localization of γ-catenin along the cell borders where cardiomyocytes contact each other.

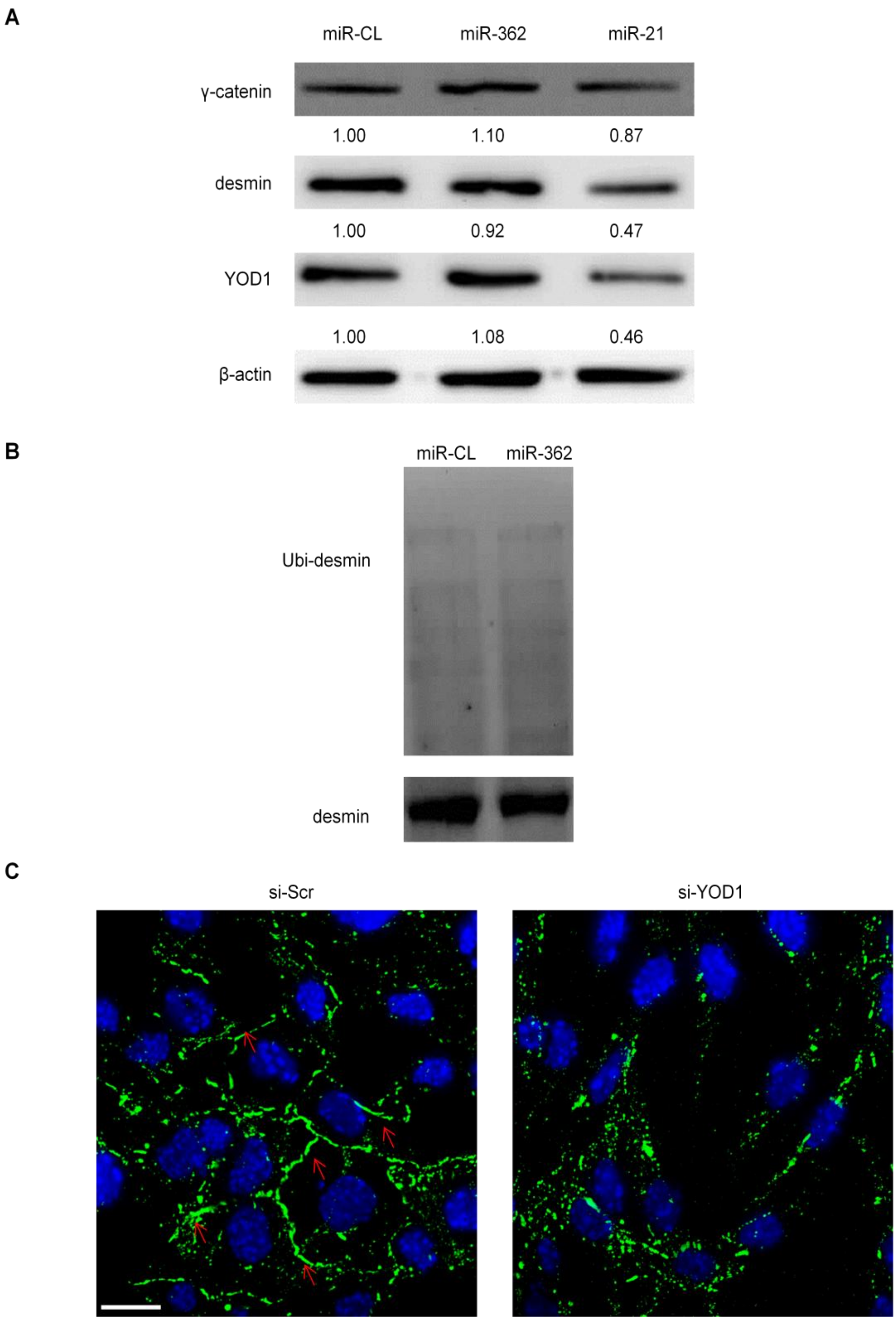

Figure S6. Knocking down of YOD1 affects γ-catenin distribution. HL-1 cells were transfected as indicated. Cellular proteins were collected for detection of γ-catenin, desmin and YOD1. The intensities of the bands were measured by using ImageJ and the signal ratios were listed below **(A)**. Desmin ubiquitination was measured using proteins pulled down by an anti-desmin antibody **(B)**. γ-catenin distribution was detected by immunofluorescence staining (green) **(C)**. Nuclei were stained with DAPI (blue). Red arrows label the localization of γ-catenin along the cell borders where cardiomyocytes contact each other.

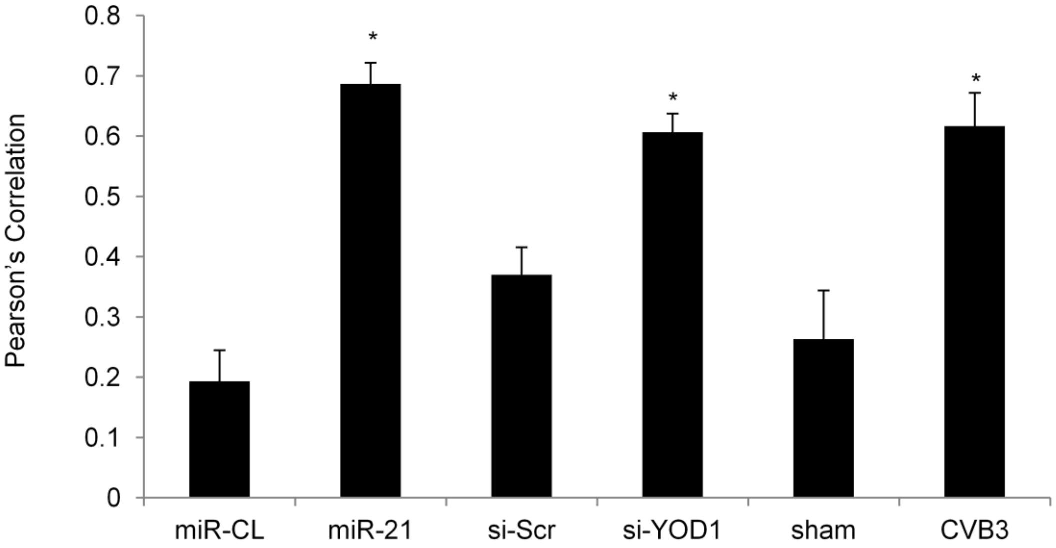

Figure S7. Pearson's Correlation analysis of co-localization of desmin proteins with proteasomes induced by miR-21, YOD1 siRNA or CVB3 infection. HL-1 cells were transfected or infected as indicated. Cells were subjected to immunofluorescence detection of desmin and proteasome (Figure 7). The co-localization of desmin and proteasome was determined by using Volocity program and Pearson's Correlation analysis. $p < 0.05$, n=4

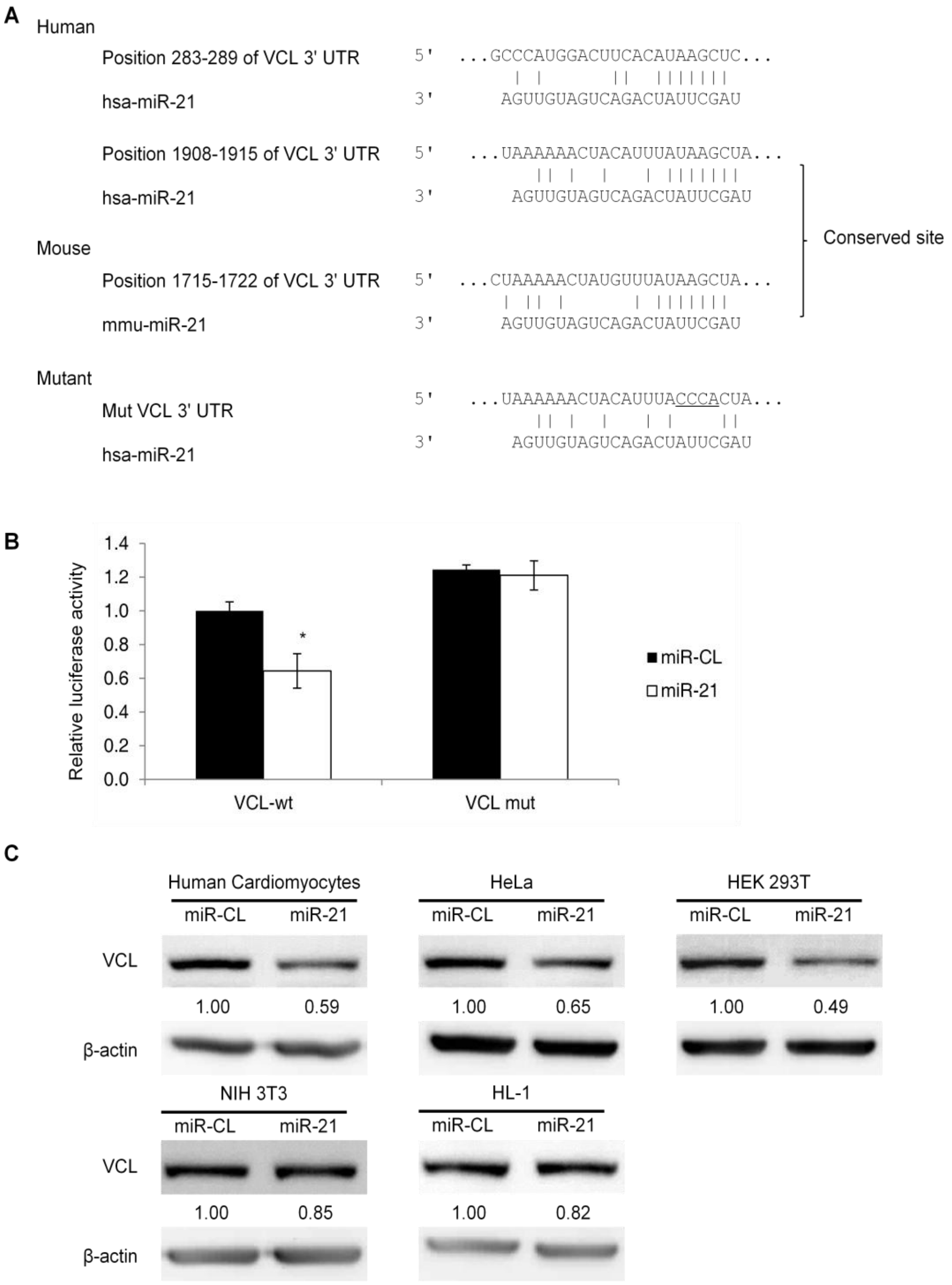

Figure S8. miR-21 targets VCL. (A) Wt and mut miR-21 targets within the VCL mRNA. The target sites within the 3’UTR of VCL mRNA of human and mouse were predicted by using the TargetScan program. A mutant site was designed for the control of luciferase assay. **(B)** Luciferase assay. HeLa cells were co-transfected with miRNA mimics and luciferase reporter plasmids harboring wt or mut VCL 3’UTR fragments. Dual luciferase assay was conducted to compare the relative luciferase activities (Firefly/Renilla) among different groups. $p < 0.05$, n=4. **(C)** miR-21 suppresses VCL in human cells but not in mouse cell lines. Different human and mouse cell lines were transfected with miR-21 and then the VCL levels were measured by WB.

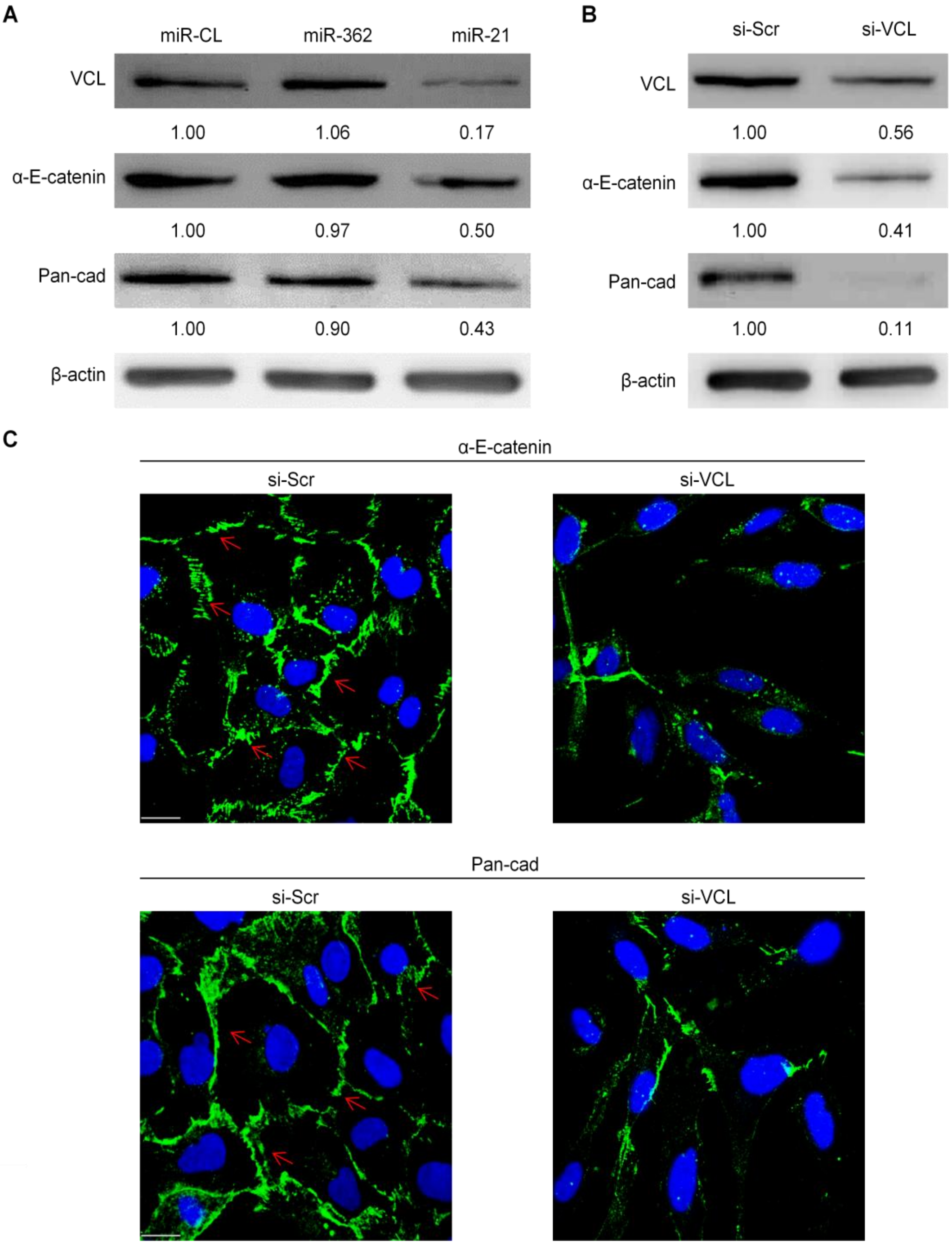

Figure S9. Knocking down of VCL interrupts fascia adherens. HL-1 cells were transfected as indicated. VCL and other proteins involved in fascia adherens were detected by WB **(A and B)**. Distributions of pan-cadherin and α-E-catenin were determined by immunofluorescence staining and confocal microscopy **(C)**. Red arrows indicate the localization of pan-cadherin or α-E-catenin along the cell borders where cardiomyocytes contact each other.

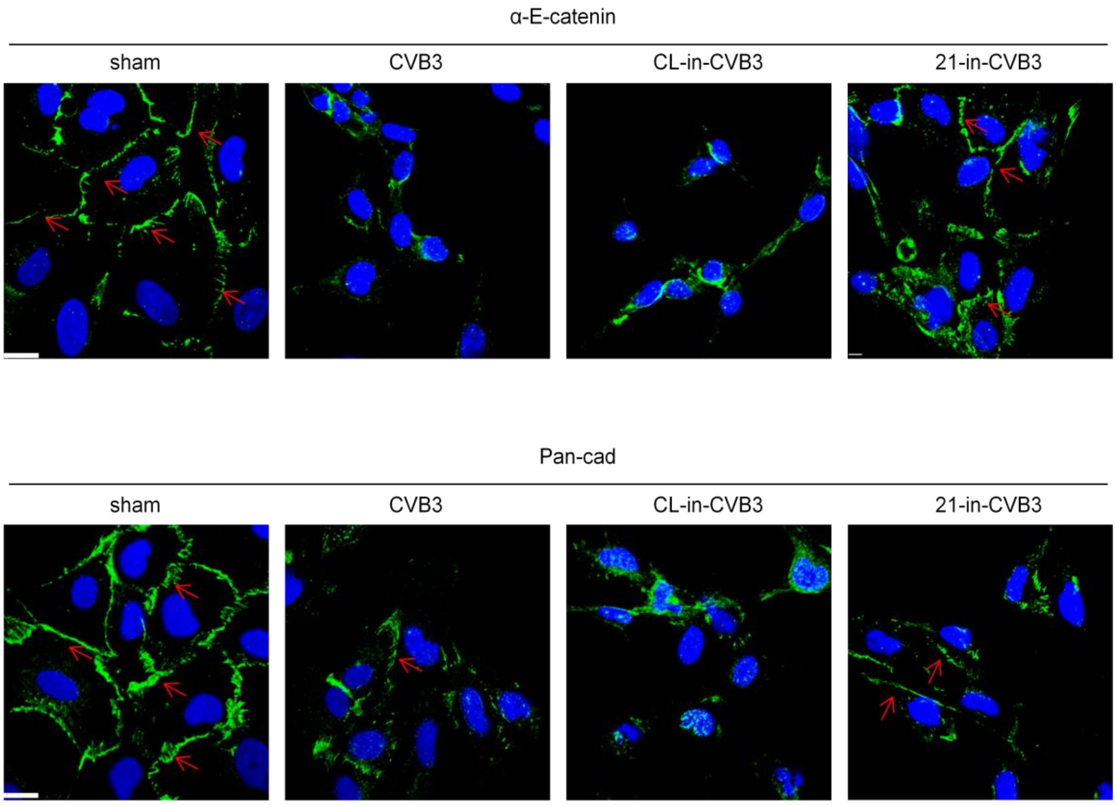

Figure S10. Knocking down of miR-21 rescues fascia adherens during CVB3 infection. HL-1 cells were transfected and infected as indicated. Distributions of pan-cadherin and α-E-catenin were determined by immunofluorescence staining and confocal microscopy. Red arrows indicate the localization of pan-cadherin or α-E-catenin along the cell borders where cardiomyocytes contact each other.

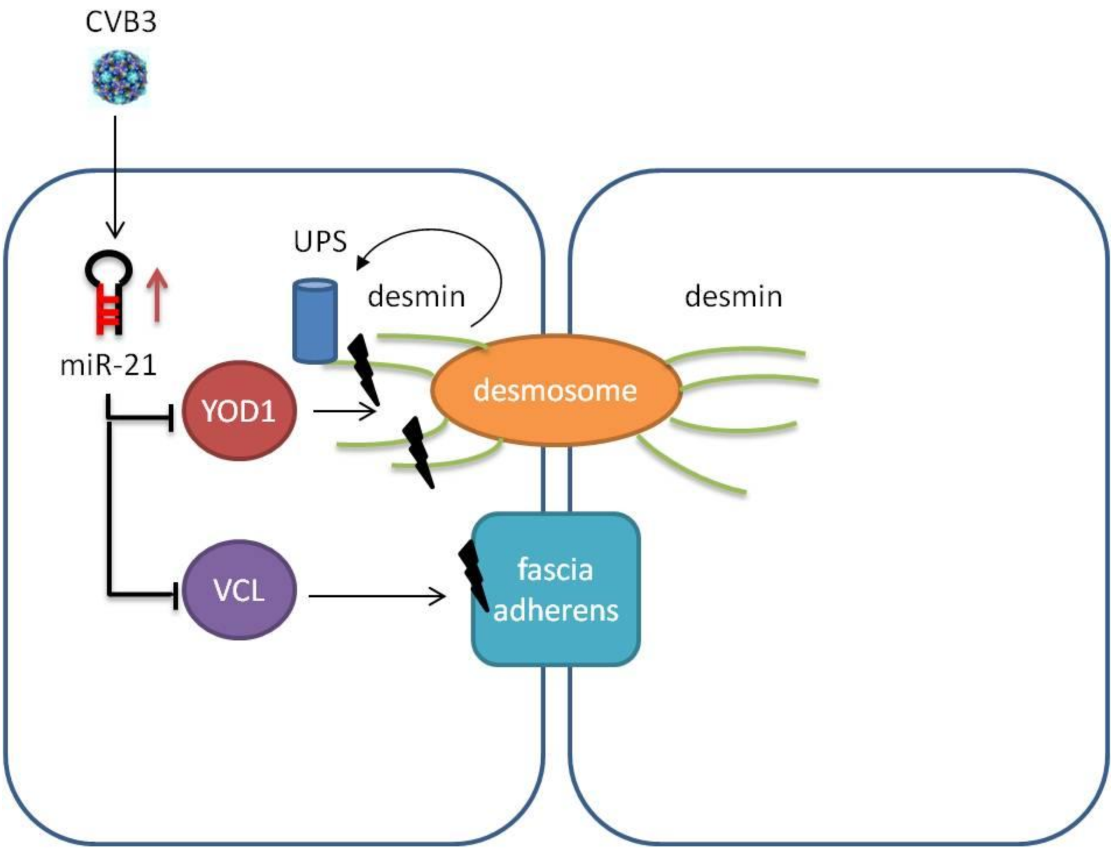

Figure S11. A putative model of miR-21 regulation on ICD integrity during CVB3 infection. CVB3 infection induces miR-21 upregulation, leading to suppression of YOD1 and VCL expression. YOD1 suppression causes desmin degradation through UPS, leading to destabilization of desmosomes. Direct targeting of VCL by miR-21 disrupts fascia adherens.

Supplementary Tables

Table S1. Top 10 potential targets of miR-21 predicted by using TargetScan

Rank	Target gene	Representative transcript	Gene name	Total context+ score
1	ZNF367	NM_153695	zinc finger protein 367	-0.76
2	GPR64	NM_001079858	G protein-coupled receptor 64	-0.57
3	YOD1	NM_018566	YOD1 OTU deubiquinating enzyme 1 homolog (S. cerevisiae)	-0.49
4	PHF14	NM_014660	PHD finger protein 14	-0.49
5	PLEKHA1	NM_001001974	pleckstrin homology domain containing, family A (phosphoinositide binding specific) member 1	-0.46
6	PIKFYVE	NM_015040	phosphoinositide kinase, FYVE finger containing	-0.45
7	PBRM1	NM_018165	polybromo 1	-0.45
8	GATAD2B	NM_020699	GATA zinc finger domain containing 2B	-0.44
9	SCML2	NM_006089	sex comb on midleg-like 2 (Drosophila)	-0.44
10	VCL	NM_003373	vinculin	-0.44

Table S2. Oligomers used for cloning and PCR

Oligo name	Sequence 5'-3'
YOD1-wt-F	AAACAAAGGAACACTTTATTTGAATAAGCTAGTTTGTT
YOD1-wt-R	CTAGAACAAACTAGCTTATTCAAATAAAGTGTTCCTTTGTTT
YOD1-mut-F	AAACAAAGGAACACTTTATTTGAACCCACTAGTTTGTT
YOD1-mut-R	CTAGAACAAACTAGTGGGTTCAAATAAAGTGTTCCTTTGTTT
VCL-wt-F	AAACCGCTAAAAAACTACATTTATAAGCTAGGATTTGTT
VCL-wt-R	CTAGAACAAATCCTAGCTTATAAATGTAGTTTTTTAGCGGTTT
VCL-mut-F	AAACCGCTAAAAAACTACATTTACCCACTAGGATTTGTT
VCL-mut-R	CTAGAACAAATCCTAGTGGGTAAATGTAGTTTTTTAGCGGTTT
Desmin-F	GTTTCAGACTTGACTCAGGCAG
Desmin-R	TCTCGCAGGTGTAGGACTGG
GAPDH-F	AGGTCGGTGTGAACGGATTTG
GAPDH-R	TGTAGACCATGTAGTTGAGGTCA

Oligos for 3'UTR cloning were flanked by PmeI and XbaI restriction enzyme sites (underlined). Mutation regions were shadowed.

Supplemental Experimental Procedures

Viral infection

CVB3 (Kandolf strain) was produced from a full-length cDNA clone (provided by Dr. Reinhard Kandolf, University of Tubingen) and propagated in HeLa cells. Virus stocks were aliquoted and stored at -80 ℃. Each batch of virus stock was titrated at the beginning of each experiment by plaque assay. Cells were seeded into 6-well plates one day before infection. Transfected cells were infected at 48 h post transfection. Cells were washed with PBS twice before incubating with the viruses diluted in serum free medium. After one hour of incubation with viruses, cells were washed with PBS twice again and supplemented with a complete culture medium. Sham infection (negative control) was performed by adding a same volume of non-infected HeLa cell culture supernatant. HL-1 cells were infected at 10 MOI (multiplicity of infection) of CVB3. Immortalized human cardiomyocytes were infected at 50 MOI. For animal infection, mice were injected intraperitoneally with 5×10^3 pfu of CVB3 in 0.2 mL of DMEM. For group size in microarray analysis, three mice were used for each group (sham or CVB3, 4 dpi or 7 dpi). For q-RT-PCR and WB analysis, five mice were used for each group.

Western blot

Tissues were rinsed with PBS to remove blood and lysed in RIPA lysis buffer (50 mM Tris-HCl (pH 7.4), 150 mM NaCl, 1 mM EDTA, 1 % Triton X-100, 0.1 % SDS, 1 % sodium deoxycholate, 1 mM PMSF, and protease inhibitor cocktail) using TissueLyser LT (Qiagen). Cells were washed with PBS and lysed in RIPA buffer as well. Samples were briefly sonicated at 40 Hz for 30 seconds and centrifuged at 13,000 × g for 20 min to collect supernatants. Protein concentration was determined by the Bradford assay (Bio-Rad) using BSA as the standard. Equal amounts of proteins were separated by SDS-PAGE with different concentrations of gels according to the size of the interested proteins and then transferred to nitrocellulose membranes (Pall Corporation). The membrane was blocked with 5% skim milk in TBST buffer (25 mM Tris-HCl, 137 mM NaCl, 0.1% (v/v) Tween-20, pH 7.6) for 1 h, incubated with a primary antibody at 4 ℃ overnight and then probed with a corresponding secondary antibody at room temperature for 1 h. The signals were then detected by using ECL reagents (Thermo Scientific). β-actin was detected for signal normalization. The primary antibodies used for WB are VP-1 (DAKO), β-Actin (Sigma), Desmin (Cell Signaling Technology), γ-catenin (Cell Signaling Technology), YOD1 (Aviva Systems Biology), Pan-caderin (Cell Signaling Technology), α-E-catenin (Cell Signaling Technology). Goat anti-mouse or Goat anti-rabbit secondary antibodies were purchased from Santa Cruz. Signal intensities were quantified by using ImageJ and normalized to the corresponding controls (set as 1.00).

Immunofluorescence staining and confocal microscopy

For immunofluorescence staining, primary antibodies for desmin (1:200) (Cell Signaling Technology), γ-catenin (1:400) (Cell Signaling Technology), α-E-catenin (1:100) (Cell Signaling Technology), pan-caderin (1:100) (Cell Signaling Technology) and anti-mouse 20S proteasome α1, 2, 3, 5, 6, & 7-subunits (MCP231) antibody (1:100) (Millipore) were used.

Alexa Fluor 488 goat anti-rabbit IgG and Alexa Fluor 594 goat anti-mouse IgG from Life Technologies were used as the secondary antibodies (1:300).

Transfection of miRNA mimics, siRNAs and miRNA inhibitors

miRNA mimics (Ambion Pre-miR miRNA Precursors, negative control #1 and miR-21), and miR inhibitors (Ambion Anti-miR miRNA Inhibitors, negative control #1 and miR-21 inhibitor) were obtained from Life Technologies. siRNAs (ON-Target Plus Non-targeting pool (scrambled control) and ON-Target plus SmartPool mouse YOD1 L-057897-01-0005) were purchased from Dharmacon. VCL-siRNA and the corresponding scrambled controls were purchased from Life Technologies. Cells (~3×10^5/well) were seeded onto 6-well plates one day before transfection to reach approximately 50% confluence at the time of transfection. Transfection was conducted using Lipofectamine RNAiMax (Life Technologies) according to the manufacturer's instructions. Briefly, cells were incubated with 2 mL of transfection complexes containing 7 µL of Lipofectamine RNAiMax and 10 nM of miRNA mimics, 50 nM of miRNA inhibitors or siRNAs for 48 h.

Reporter Constructs and dual luciferase assay

pmirGLO Dual-Luciferase miRNA target expression vector was purchased from Promega. Oligomers containing miR-21 targeting sites were synthesized, annealed using oligo annealing buffer (Promega) and inserted into the 3' end of firefly luciferase gene at the PmeI and XbaI sites in the reporter vector. The vector also contains a Renilla luciferase gene serving as an internal control for transfection efficiency. The reporter constructs were co-transfected with miR-CL or miR-21 using Lipofectemine 2000 (Life Technologies) following the manufacturer's instructions. For each transfection, 150 ng/well of reporter plasmids, 2 pmol/well of miRNA mimics (20 pmol/well) and 1 µL/ well of Lipofectamine 2000 were used for 24-well plates. At 48 h post transfection, firefly and Renilla luciferase activities were measured using the Dual-Glo luciferase analysis system (Promega) according to the manufacturer's protocol. All the assays were performed in triplicate. The ratios of firefly to Renilla luciferase activity were calculated and normalized to the miR-CL control group which was set as 1.0.

Co-transfection of desmin vectors with miRNA and siRNAs

HL-1 cells were seeded at 5×10^5 cells/well in 6-well plates and co-transfected with desmin (wt or mut) plasmids and miRNAs or siRNAs using Lipofectamine 2000 according to manufacturer's instructions. Briefly, 50 pmol/well of siRNA or miRNA were mixed with 1.5 µg/well of plasmids and 9 µL/well of Lipofectamine 2000 in 250 µL Opti-MEM (Gibco). The mixture were added onto the cells and incubated with the cells for 48 h. The samples were then collected for further analysis. Cells transfected with desmin plasmids only (48 h) were infected with CVB3 at 10 MOI for 24 h for detecting desmin levels and desmin ubiquitination.
